# Characterization of L1 ORF1p Self-Interaction and Cellular Localization Using a Mammalian Two-Hybrid System

**DOI:** 10.1371/journal.pone.0082021

**Published:** 2013-12-04

**Authors:** Mark Sokolowski, Dawn deHaro, Claiborne M. Christian, Kristine J. Kines, Victoria P. Belancio

**Affiliations:** Department of Structural and Cellular Biology, Tulane School of Medicine, Tulane Cancer Center, Tulane Center for Aging, New Orleans, Louisiana, United States of America; Louisiana State University, United States of America

## Abstract

Long INterspersed Element-1 (LINE-1, L1) is an active retrotransposon that mobilizes using a ribonucleoprotein particle (RNP) intermediate composed of the full-length bicistronic L1 mRNA and the two proteins (ORF1p and ORF2p) encoded by that mRNA. ORF1p and ORF2p demonstrate *cis*-preference for their encoding mRNA. Previous studies of ORF1p, purified from bacterial and insect cells demonstrated that this protein forms trimers *in vitro*. While valuable for understanding ORF1p function, these *in vitro* approaches do not provide any information on ORF1p self-interaction in the context of mammalian cells. We used a mammalian two-hybrid (M2H) system in order to study L1 ORF1p self-interaction in human and mouse cells. We demonstrate that the M2H system successfully detects human and mouse ORF1p self-interactions in transiently transfected mammalian cells. We also generated mouse and human ORF1p-specific antibodies to characterize the expression of ORF1p fusion proteins used in the M2H system. Using these antibodies, we demonstrate that ORF1p interaction *in trans* leads to the formation of heterodimers that are expected to produce a positive signal in the M2H system. Although the role for L1 ORF1p *cis*-preference in L1 mobilization is established, the impact of ability of ORF1pto interact *in trans* on the L1 replication cycle is not known. Furthermore, western blot analysis of ORF1p generated by a full-length L1, wild type ORF1, or a codon-optimized ORF1 expression vector is detected in the nucleus. In contrast, the addition of a tag to the N-terminus of the mouse and human ORF1 proteins can significantly alter the subcellular localization in a tag-specific manner. These data support that nuclear localization of ORF1p may contribute to L1 (and potentially the SINE Alu) RNP nuclear access in the host cell.

## Introduction

Long INterspersed Element-1 (LINE-1, L1), an autonomous non-long terminal repeat retrotransposon, has contributed greatly to the evolution of the human genome through retrotransposition of itself and facilitation of retrotransposition of the parasitic SINE Alu and SVA elements [1-3]. There are roughly 500,000 related copies of L1 distributed throughout the human genome [4]. Though the majority of these loci are 5'-truncated, there are 80-100 full-length L1 copies that are predicted to retain activity [4-8]. A full-length, autonomous L1 is composed of a 5’ untranslated region (UTR) with an internal promoter, two open reading frames (ORF1 and ORF2), and a 3’ UTR ending in a polyA site and associated polyA tail [9,10]. ORF1 and ORF2 proteins (ORF1p and ORF2p) are translated from the bicistronic L1 mRNA [11] and, potentially, from prematurely polyadenylated and spliced L1 mRNAs [12,13]. Association of ORF1p, ORF2p, and the full-length L1 mRNA which generated these proteins into a ribonucleoprotein particle (RNP) is required for L1 retrotransposition [1,14]. The protein components of the L1 RNP exhibit *cis*-preference for their encoding L1 mRNA, thereby limiting the retrotransposition of cellular mRNA and L1 mRNA produced by defective L1 loci [15,16]. 

ORF1p is a 40 kDa protein with an N-terminal domain, coiled-coil domain (CCD), an RNA recognition motif (RRM) and a C-terminal domain (CTD) [17]. ORF1p exhibits RNA binding and nucleic acid chaperone properties, which are required for L1 mobilization [14,18]. Human and mouse ORF1p (hORF1p and mORF1p) trimerize through their respective CCDs and associate with their corresponding full-length mRNAs, a process that is crucial for L1 RNP formation [19-22]. It is estimated that an ORF1p trimer occupies about 50 nucleotides on the template RNA, suggesting that ORF1p trimers may be the most abundant component in the L1 RNP [20-22]. 

As part of the L1 replication cycle, the L1 RNP must enter the nucleus. While this is an essential step for L1 amplification, the mechanism of L1 nuclear entry is currently unknown. It has been suggested that L1 ORF2p plays a role in the nuclear localization of the L1 RNP [23], while any contribution of L1 ORF1p to the subcellular localization of the L1 RNP is poorly understood. ORF1p fused to a green fluorescent protein (GFP), as well as other tags, has been detected primarily in the cytoplasm by immunohistochemistry (IHC) [24]. The cytoplasmic localization of this fusion protein was affected by various truncations of the ORF1p portion of the chimeric protein [24]. Recently, endogenously expressed ORF1p has been detected by IHC in both the nuclei and cytoplasm of cells of human tumor samples collected from patients with different disease stages [25,26]. Nuclear and cytoplasmic L1 ORF1p was also reported to interact with multiple cellular proteins in both compartments [27]. These data demonstrate that the subcellular localization of ORF1p may vary depending on the cell type, and suggest the possibility that its localization may be affected by the addition of tags. While these examinations of ORF1p localization are, by necessity, carried out *in vivo*, studies dealing with ORF1p self-interaction have almost exclusively been performed *in vitro*.

The majority of the critical findings regarding ORF1p trimerization have been made *in vitro* using human or mouse proteins purified from *E. coli* or *baculovirus*-infected insect cells [18-20,22]. The ORF1p behavior *in vitro* is presumed to translate to the mammalian environment. However, limited experimental information exists concerning the properties and functions of ORF1p in mammalian cells. While the data acquired *in vitro* are invaluable, these approaches are also laborious, technically challenging, and do not account for the potential influence of host cellular factors on ORF1p self-interaction. Recently, many cellular proteins have been reported to interact with L1 ORF1p in the nucleus and cytoplasm [27,28], suggesting that cellular factors, unaccounted for in *in vitro* approaches, may influence the behavior of ORF1p. To capture L1 ORF1p self-interaction in a more biologically relevant manner, we used the mammalian two-hybrid system (M2H). A major advantage of utilizing this approach is that it recapitulates some aspects of ORF1p biology related to L1 replication cycle such as ORF1p self-interaction. From here on, the ORF1p self-interaction is defined as dimerization or trimerization of ORF1p molecules translated from the same or different mRNAs.

Our data demonstrate that the M2H system detects human and mouse ORF1p self-interactions in transiently transfected human and mouse cells. Using polyclonal antibodies specific to human and mouse ORF1p, we demonstrate that the interactions detected by the M2H system are due, at least in part to the formation of heterodimers *in trans*. ORF1p heterodimerization *in trans* or self-interaction *in trans* is defined as the interaction between ORF1 proteins made from different mRNAs. ORF1p heterodimerization occurs with various efficiencies in human and mouse cells as detected by western blot analysis and the M2H system. We also detected significant presence of ORF1p in the nucleus and demonstrate that the fusion of various tags to the N-terminus of the ORF1p alters its localization in a tag-specific manner. Nuclear localization of the untagged L1 ORF1p expressed from the full-length wild type (wt) L1 supports the theory that L1 ORF1p may assist L1 RNPs in gaining access to the nucleus. 

## Results

### Mammalian two-hybrid system detects human and mouse ORF1p self-interaction

**Figure 1:**
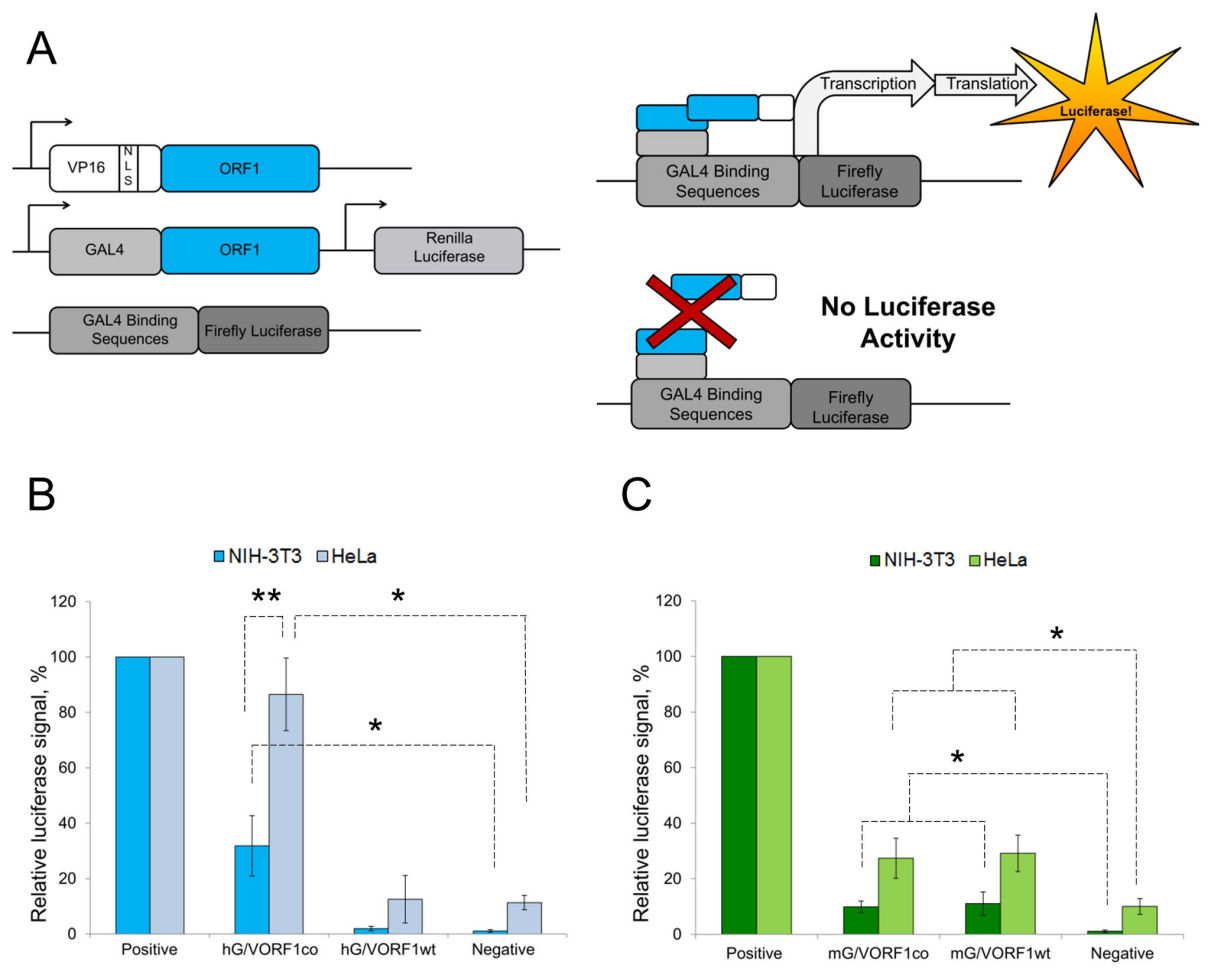
Analysis of ORF1p interaction using the M2H system. **A**. (left) Schematic of the M2H constructs, arrows denote promoters. One ORF1p is fused to a GAL4 binding protein, derived from the GAL4 yeast transcription factor which binds a specific sequence of DNA. The other ORF1 protein is fused to the VP16 viral transcriptional transactivator protein sequence, with an added SV40-derived internal nuclear localization signal. Tandemly-arrayed GAL4 binding sites are present upstream of the firefly luciferase gene. (top right) Interaction between proteins of interest expressed from the M2H expression plasmids leads to firefly luciferase expression. (bottom right) If the proteins do not interact with one another, no firefly luciferase is produced. **B**. M2H results for codon-optimized and wild type hGORF1p:hVORF1p interaction in HeLa and NIH-3T3 cells normalized to the positive control. Asterisk denotes statistical significance between the relative luciferase signals detected for hG:VORF1p interaction (wt or co) in HeLa or NIH-3T3 cells compared to their respective negative control (p-value <0.05). Double asterisk denotes statistical significance between relative signal reflecting interaction between hORF1co and VORF1co detected in HeLa versus NIH-3T3 cells (p-value<0.05). **C**. M2H results for codon-optimized and wild type mGORF1p:mVORF1p interactions in HeLa and NIH-3T3 cells normalized to their respective positive controls. Asterisk denotes statistical significance between the relative luciferase signals detected for mG:VORF1p (wt or co) when compared to their respective negative controls (p-value<0.05).

We used the mammalian two-hybrid system (M2H) to assess ORF1p self-interaction in transiently transfected mammalian cells. The M2H system utilizes three expression vectors that allow for testing interactions between two specific proteins (Figure 1A) which are fused to either a GAL4 DNA-binding domain or a VP16 transcriptional activation domain. The GAL4 binding domain of the interacting pair is expected to bring cellular transcription machinery recruited by the VP16 activation domain to a reporter plasmid containing GAL4 binding sites upstream of a promoterless firefly luciferase gene. Therefore, luciferase activity is detected only if the GAL4- and the VP16-fused proteins stably interact in mammalian cells (Figure 1A). The positive control used in both HeLa and 3T3 cell lines is the interaction between MyoD, a myogenic regulatory protein, and Id, a negative regulator of myogenic differentiation. The negative control is GAL4-fused ORF1 codon-optimized (GORF1co) cotransfected with untagged ORF1co. Both human and mouse ORF1p trimerize and multimerize *in vitro* [19,20]. If these interactions are preserved in the mammalian environment, the M2H system is expected to detect interactions between ORF1 GAL4 and VP16 homotrimers or monomers, provided that ORF1p can interact *in trans*. ORF1p interactions *in trans* are defined as interactions between two or more ORF1 proteins generated from different mRNAs.

We assessed human ORF1p self-interactions using the M2H system by subcloning codon-optimized ORF1 sequences [29,30] into the GAL4 and VP16 expression vectors (see material and methods and Table S1). Transient transfection of HeLa cells with human codon-optimized GAL4- and VP16-ORF1 fusion expression plasmids (hGORF1co and hVORF1co) detected robust ORF1p self-interaction (Figure 1B). Weaker human ORF1p self-interaction was detected in mouse NIH-3T3 cells as compared to human cells (Figure 1B). Analysis of luciferase activity in cells transiently transfected with GAL4- and VP16-fused codon-optimized mouse ORF1 expression plasmids [30] (mGORF1co and mVORF1co, respectively) demonstrated that HeLa cells supported more efficient self-interaction of mouse ORF1p than did NIH-3T3 cells (Figure 1C, mG/VORFco). In all experiments, Renilla luciferase generated from the GORF1 expressing plasmid was used to normalize for transfection efficiency. 

To determine whether self-interaction of human and mouse ORF1 proteins produced by wild type (hORF1wt and mORF1wt) rather than codon-optimized L1 ORF1 sequences can be detected by the M2H system, we subcloned wt human and wt mouse ORF1 sequences into the M2H expression vectors. Figure 1C demonstrates that mouse ORF1p produced from the wild type ORF1 sequence can self-interact as efficiently as the protein generated from the plasmid containing codon-optimized ORF1 sequence when transiently transfected in HeLa cells (Figure 1C, mG/VORF1wt and mG/VORF1wt). However, there was no self-interaction detected for the human ORF1p generated from the wt expression plasmid in either cell line (Figure 1B). 

We also tested the possibility that mouse and human ORF1p may interact with one another in mammalian cells. Cotransfection of M2H plasmids expressing hORF1co and mORF1co fusion proteins in HeLa and NIH-3T3 cells did not produce any luciferase signal, demonstrating the species specificity of ORF1p interactions (Figure S1). These data demonstrate that the M2H system successfully detects specific ORF1p self-interactions in mammalian cells. This system complements existing *in vitro* methods and provides a unique alternative for testing ORF1p interactions. 

### ORF1 protein expression in human and mouse cells

**Figure 2:**
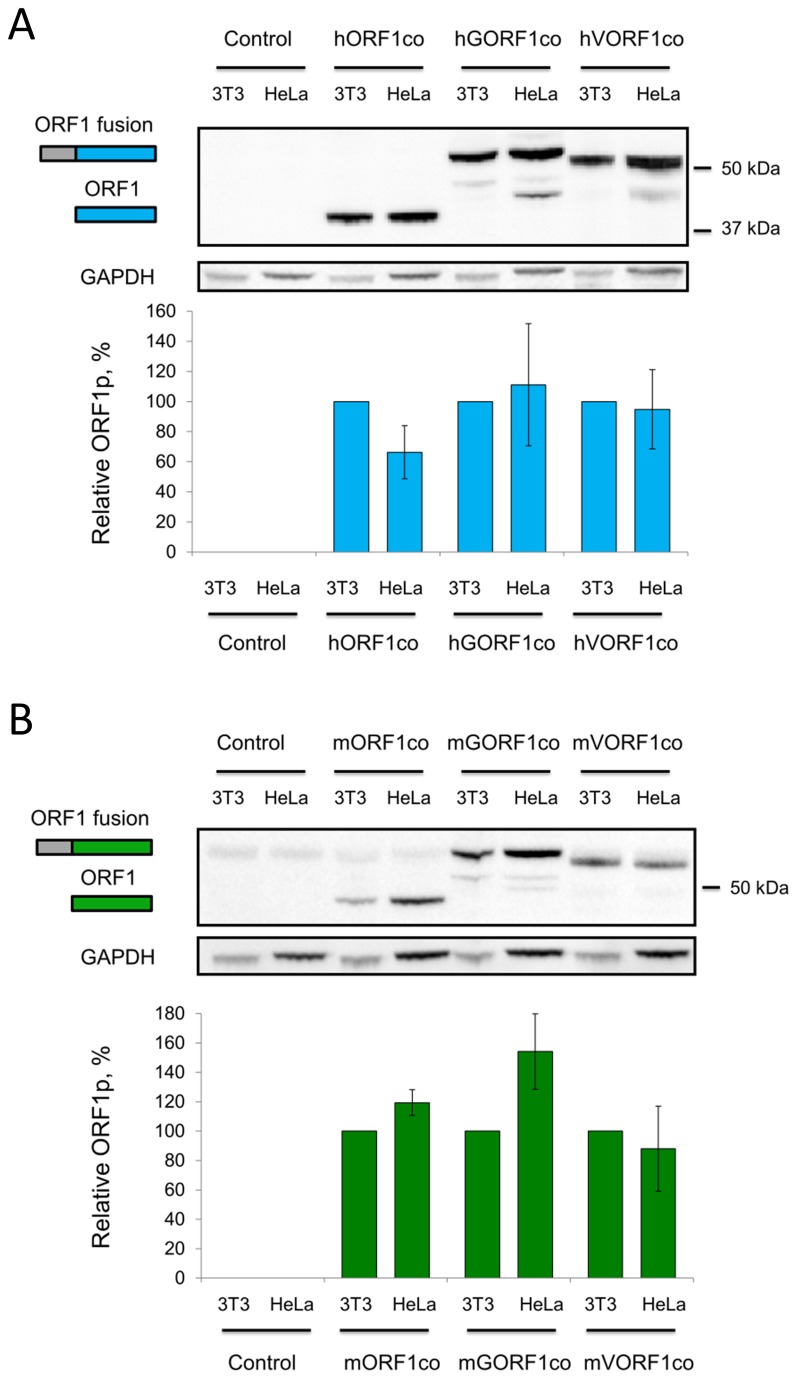
Analysis of M2H ORF1 fusion protein expression in human and mouse cells. **A**. (top) Western blot analysis of codon-optimized human ORF1 (hORF1co, 40 kDa, blue rectangle labeled as ORF1) and human ORF1co N-terminally fused to GAL4 or VP16, (hGORF1co, 57 kDa, and hVORF1co, 49 kDa, blue and grey rectangles labeled as ORF1 fusion) transiently transfected in HeLa or NIH-3T3 cells. Human ORF1 protein was detected with custom-made human-specific ORF1 polyclonal antibodies. GAPDH is used as a loading control. 37 and 50 kDa are molecular markers. Control lanes indicate cells transfected with an empty vector. (bottom) Quantitation of western blot results. Signals obtained for hORF1p and its fusions were normalized to their respective GAPDH loading controls and expressed as a percentage of the relative signal detected for each protein in NIH-3T3 cells. **B**. (top) Western blot analysis of codon-optimized mouse ORF1 (mORF1co, 47 kDa, T7-Myc-His tag, green rectangle labeled as ORF1) and mouse ORF1co N-terminally fused to GAL4 and VP16 (mGORF1co, 60 kDa, and mVORF1co, 52 kDa, green and grey rectangles labeled as ORF1 fusion) transiently expressed in HeLa or NIH-3T3 cells. 50 kDa is a molecular marker (bottom) Quantitation was performed as described in A.

The difference in relative ORF1p self-interactions observed between human and mouse cells as well as the lack of detectable self-interaction between the human ORF1 fusion proteins generated from wt plasmids prompted the investigation of expression of the ORF1 fusion proteins in these cell lines. To accomplish this analysis, we generated human and mouse L1 ORF1p-specific antibodies (Figure S2A and S2B). Western blot analysis of human ORF1p produced by the plasmids containing codon-optimized ORF1 sequence (hORF1co, expected size 40 kDa), GAL4- (hGORF1co, expected size 57 kDa) or VP16-ORF1co (hVORF1co, expected size 49 kDa) sequences demonstrated similar steady-state levels of total protein for each of these constructs in both human and mouse cells (Figure 2A). A similar result was observed for the respective mouse proteins, mORF1co (expected size 47 kDa due to a T7-Myc-His tag), mGORF1co (expected size 60 kDa), mVORF1co (expected size 52 kDa) (Figure 2B), demonstrating that the overall protein expression did not account for the differences detected by the M2H system (Figure 1B and 1C). 

Western blot analysis of the total ORF1 protein generated by the M2H expression plasmids containing wt human L1 ORF1 sequences, hGORFwt (expected size 57 kDa) and hVORFwt (expected size 49 kDa), did explain the lack of signal from these expression plasmids in the M2H assay (Figure S3A). No detectable protein was produced by either one of these plasmids, even when transfection conditions were manipulated in an attempt to increase the signal (data not shown). In contrast, the unfused human ORF1p expressed by the full-length wt L1 (hL1wt, expected size 40 kDa) or ORF1 expression plasmids (hORF1wt , expected size 41 kDa due to a T7 tag, hORF1co, expected size 40 kDa) was readily detectable in HeLa cells (Figure S2A). We speculate that our inability to detect human fusion proteins generated from wt plasmids can be explained by splicing between L1 splice sites and an intron present in the M2H expression vectors [13,31,32]. Western blot analysis of ORF1 protein generated by the wt mouse L1 ORF1 sequences subcloned into the M2H expression vectors detected mouse GAL4 and VP16 fusion proteins in HeLa cells (Figure S3B, nuclear fraction). However, the steady-state levels of mVORF1wt were significantly reduced in comparison to the mGORF1wt protein. Western blot analysis of the corresponding mouse proteins demonstrate that mGORF1wt, but not mVORF1wt, is expressed in NIH-3T3 cells (Figure S3C), providing an explanation for the difference in the M2H signal between the two cell lines (Figure 1C).

### Subcellular localization of human and mouse ORF1 proteins

**Figure 3:**
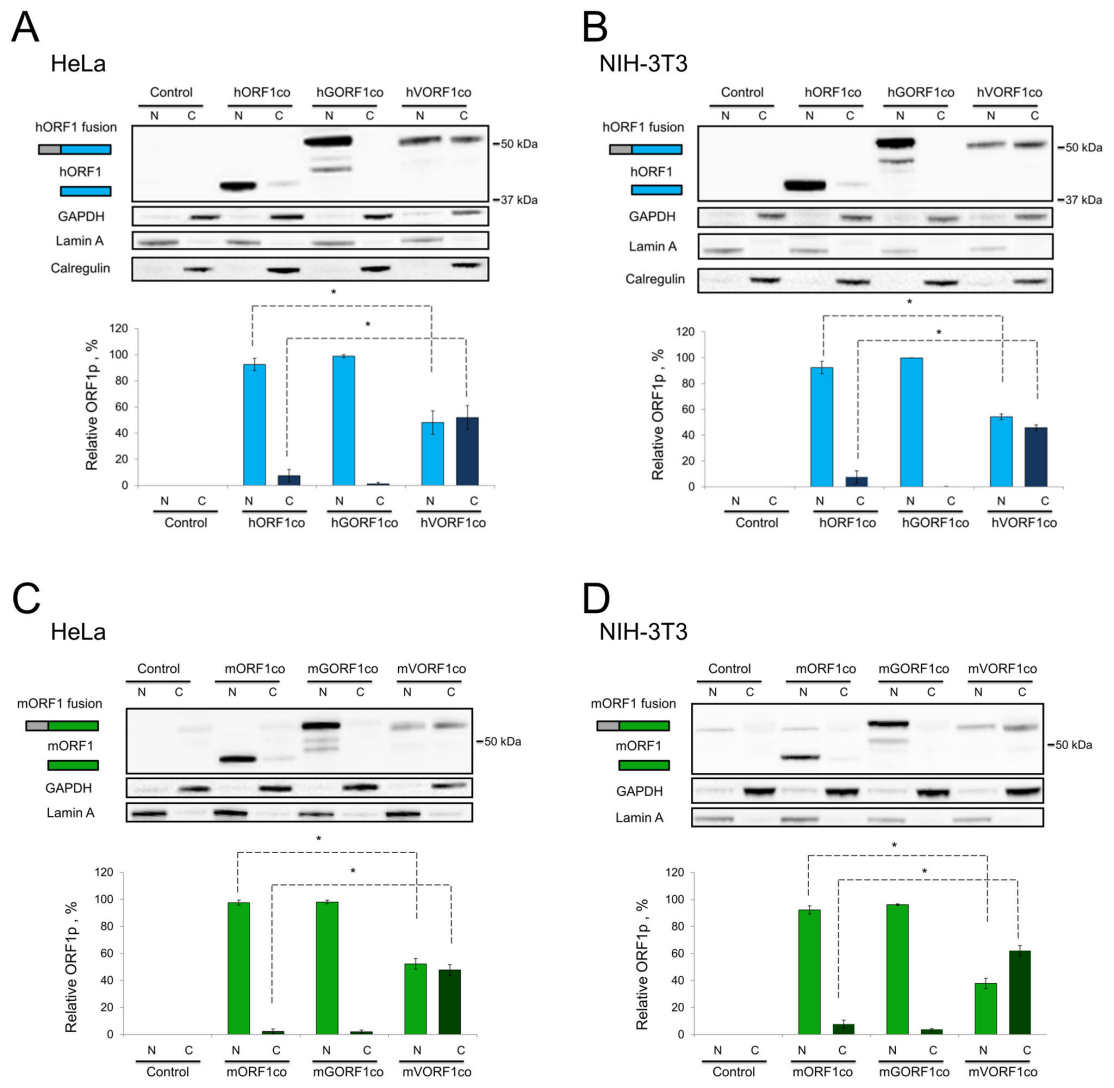
Analysis of subcellular localization of ORF1 protein fusions in human and mouse cells. **A**. (top) Western blot analysis of subcellular localization of hORF1co (blue rectangle labeled as hORF1), hGORF1co and hVORF1co (grey/blue rectangle labeled as hORF1 fusion) transiently expressed in HeLa cells. Subcellular localization of the human ORF1 protein detected in nuclear (N) and cytoplasmic (C) fractions with human-specific ORF1p polyclonal antibodies. GAPDH (cytoplasmic marker), Lamin A (nuclear marker), and Calregulin (endoplasmic reticulum marker) proteins are used as loading and cell fractionation controls. Control lanes indicate cells transfected with empty vector. (bottom) Quantitation of western blot results. hORF1 and its fusions were normalized to their respective GAPDH and Lamin A loading controls. The relative nuclear and cytoplasmic ORF1p was calculated as a fraction of the relative total ORF1p detected in respective cell types. **B**. The same experiment and analysis as in A, but using NIH-3T3 cells. **C**. Western blot analysis as above using mORF1co (green rectangle labeled as mORF1), mGORF1co or mVORF1co (grey/green rectangle labeled as mORF1 fusion) in HeLa cells. Mouse specific ORF1p polyclonal antibodies were used for protein detection. **D**. The same experiment and analysis as in C, using NIH-3T3 cells. For all panels, statistically significant data points are indicated by dashed lines. An asterisk represents a significant difference between the subcellular localization of ORF1p and VP16-fused ORF1p (T-test, p-value <0.05).

While the total steady-state protein levels of GAL4- and VP16-fused human ORF1 generated from codon-optimized expression plasmids do not differ between NIH-3T3 and HeLa cells, the subcellular localization of these fusion proteins may vary between the cell lines and potentially have an impact on the interactions required to produce a signal in the M2H system. We performed nuclear/cytoplasmic fractionation of HeLa and NIH-3T3 cells transiently transfected with codon-optimized expression plasmids generating untagged, GAL4-, or VP16-fused ORF1p. Western blot analysis demonstrated that untagged hORF1co and GAL4-fused hGORFco are detected predominantly in the nucleus of both cell types (Figure 3A and B). In contrast, a much higher proportion of the VP16-fused ORF1p (i.e., hVORF1co *vs.* hORF1co) is detected in the cytoplasmic fractions of HeLa and NIH-3T3 cells (Figure 3A and B), despite the fact that the VP16 fusion ORF1p contains a nuclear localization signal (NLS). The quantification of western blot results was done by normalization of the ORF1p signal in each fraction to its respective loading controls (GAPDH and Lamin A). The relative nuclear and cytoplasmic ORF1p was calculated as a percent of the combined nuclear and cytoplasmic ORF1p detected in each cell type (Figure 3A and B). Western blot analysis of the respective tagged and untagged mouse ORF1 proteins demonstrated the same pattern of subcellular distribution as was detected for the human ORF1p (Figure 3C and 3D). These results demonstrate that ORF1p subcellular localization can be significantly altered by the addition of an N-terminal tag.

**Figure 4:**
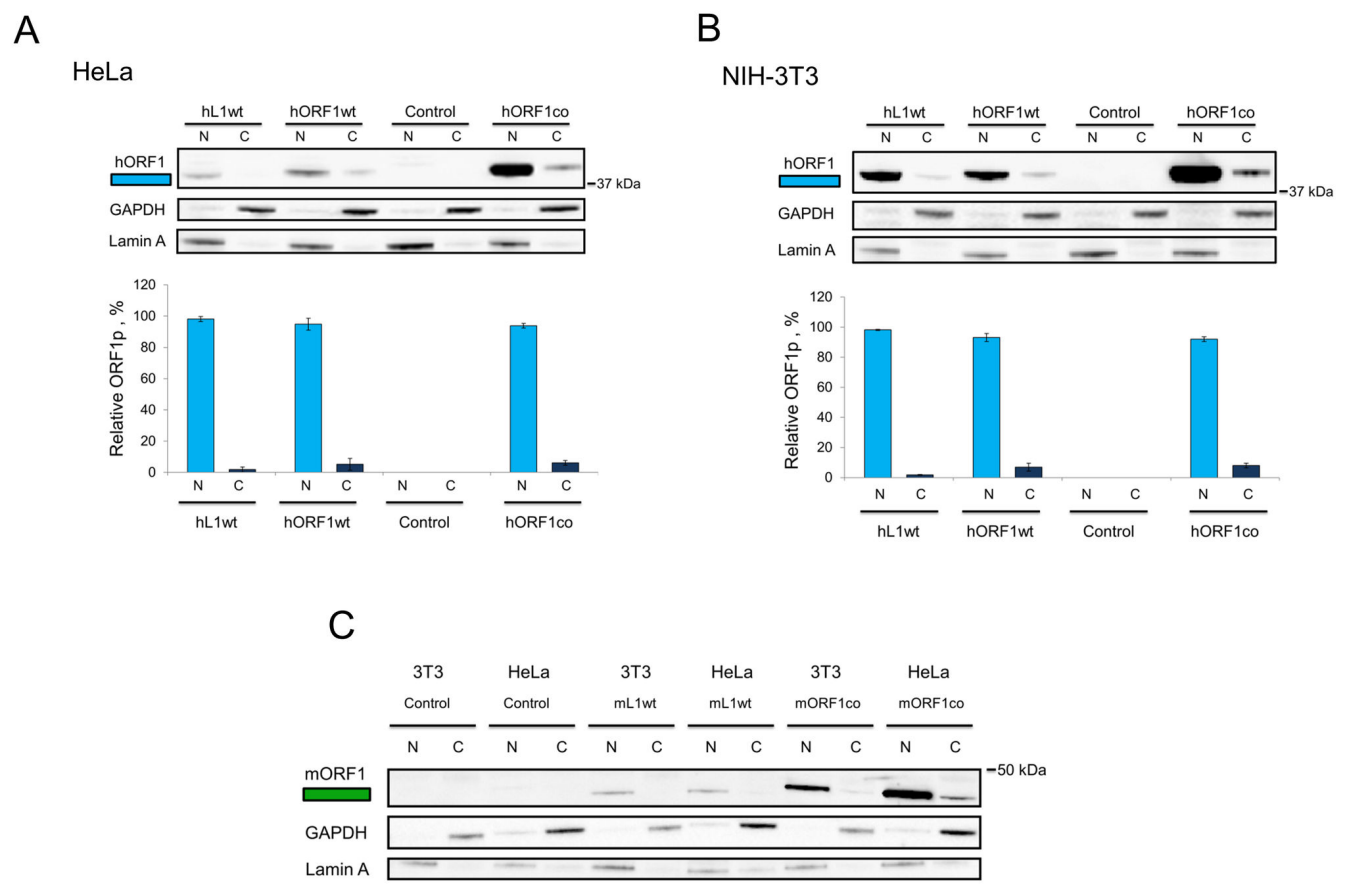
Analysis of subcellular localization of hORF1p expressed from vectors containing wild type sequences. **A**. (top) Western blot analysis of subcellular localization of ORF1p (blue rectangle labeled as hORF1) expressed from human full-length L1 (hL1wt, 40 kDa), human ORF1 wt (hORF1wt, 41 kDa) or hORF1co (40 kDa) vectors transiently expressed in HeLa cells. Human ORF1p is detected in nuclear (N) and cytoplasmic (C) fractions with human-specific ORF1p polyclonal antibodies. GAPDH (cytoplasmic marker) and Lamin A (nuclear marker) are used as loading and cell fractionation controls. 37 kDa is a molecular marker. Control lanes indicate cells transfected with empty vector. (bottom) Quantitation of western blot results: each construct expressing hORF1p was normalized to its respective GAPDH and Lamin A loading controls. The relative amounts of nuclear and cytoplasmic ORF1p were calculated as a fraction of the relative total ORF1p detected. No statistical significance was found between the subcellular localization of ORF1p expressed by the above-described constructs. **B**. The same experiment and analysis as in A was performed in NIH-3T3 cells. No statistical significance was found between the subcellular localization of ORF1p expressed by the above-described constructs. **C**. Subcellular localization of mORF1p (green rectangle labeled as mORF1) transiently expressed from the full-length mouse L1 (mL1wt) or codon-optimized mORF1 (mORF1co) in HeLa or NIH-3T3 cells using mouse specific polyclonal antibodies. 50 kDa is a molecular marker.

Human ORF1p C-terminally-fused to a green fluorescent protein (ORF1-GFP) has been previously reported to localize predominantly to the cytoplasm when detected by immunohistochemistry (IHC) [24]. Recently, endogenous L1 ORF1p was detected by IHC in the nuclei of human tumor samples [25,26], suggesting that the presence of a tag or the cell type may influence subcellular localization of the ORF1 protein. Figure 3 demonstrates that human and mouse ORF1 proteins expressed from the plasmids containing codon-optimized L1 ORF1 sequence predominantly localize to the nucleus, as do the GAL4-fused versions of both proteins (hGORF1co and mGORF1co), in both HeLa and NIH-3T3 cells. In contrast, both human and mouse ORF1 proteins fused to VP16 were found in the nucleus and cytoplasm of HeLa and NIH-3T3 cells (Figure 3, hVGORF1co and mVORF1co). To rule out any potential influence of codon-optimization on the subcellular localization of ORF1p, we examined the subcellular localization of ORF1 proteins expressed from plasmids containing wt L1 and ORF1 sequences. Figures 4A and 4B demonstrate that ORF1p expressed by the plasmid containing wild type ORF1 sequence or by the wild type full-length human L1 is found predominantly in the nuclear fraction of human (HeLa and PC3, Figure S4A) and mouse (NIH-3T3) cells (Figure 4C). The plasmid containing wt ORF1 sequence expresses an ORF1p that is C-terminally fused to a T7 tag. Our data demonstrate that the T7 tag does not alter ORF1p subcellular localization consistent with the report that this tagged ORF1p supports L1 retrotransposition [15]. Similarly, mouse ORF1p transiently expressed by the wild type full-length L1 (mL1wt) and mORF1co expression plasmids demonstrated preferential nuclear localization in both human and mouse cells (Figure 4C, mL1wt and mORF1co, respectively). 

Figures 3 and 4 show western blot results obtained by comparing equal amounts of protein collected from nuclear and cytoplasmic fractions. This approach does not take into account the fact that these total amounts represent different percentages of the total protein harvested from each compartment. Loading equal percentages of the total protein harvested from each compartment shows that hORF1p is still predominantly detected in the nuclear fraction of transiently transfected cells (Figure S4B).

It is possible that the time elapsed between transfection and protein harvest, as well as the intracellular concentrations of ORF1p, could affect its subcellular localization. ORF1p produced from the full-length human L1.3 expression vector was detected predominately in the nucleus as early as 12 hours after transfection (Figure S4C). This finding supports that ORF1p nuclear localization is present as soon as the protein has reached detectable levels. Western blot analysis of nuclear and cytoplasmic fractions from HeLa cells transiently transfected with increasing amounts of codon-optimized human ORF1 expression plasmid further demonstrated that the nuclear localization of L1 ORF1p is consistent within the experimental range (Figure S4D). 

To further eliminate the influence of methodological artifacts on the ORF1p subcellular localization, we tested different processing and centrifugation conditions for nuclear/cytoplasmic fractionation (Figure S4E), all of which resulted in ORF1p detection in the nucleus. These data support our observation that the difference in the subcellular localization of the human and mouse VORF1 proteins, when compared to the unfused ORF1p appears to be tag-specific, as both human and mouse GAL4-ORF1 fusion proteins retain their nuclear localization (Figures 3 and 4). This tag-specific effect on L1 ORF1p localization should be taken into consideration when conclusions about L1 ORF1p biology are drawn from the studies of tagged proteins. 

### Heterodimer formation by the ORF1 proteins expressed from different mRNAs (heterodimerization *in trans*)

Our above-described approaches eliminated differing protein levels and subcellular localization as reasons contributing to the difference in the hORF1p self-interactions detected using the M2H assay in human and mouse cells (Figure 1). To identify the reason for this observed difference, we next determined which type of ORF1p interaction is responsible for the signal detected by the M2H system. Interactions between ORF1p homotrimers [20] as well as ORF1p self-interaction *in trans* could lead to transcriptional activation and expression of the reporter gene in the M2H system. 

**Figure 5:**
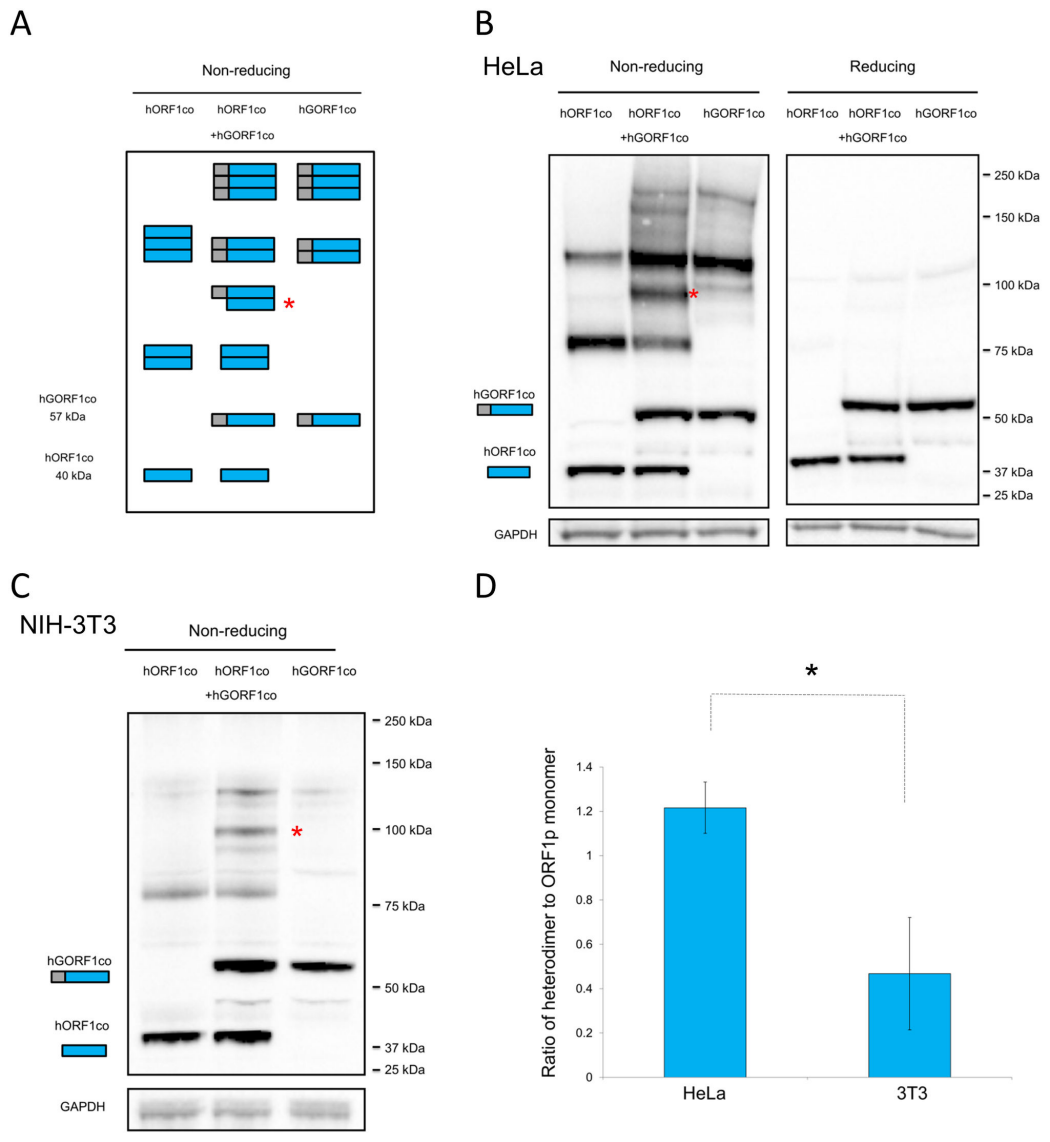
Analysis of ORF1p interactions *in* *trans*. **A**. Schematic of predicted ORF1p species expected to be detected if ORF1p heterodimerization takes place when cells are transiently cotransfected with tagged and untagged ORF1p expression plasmids. Blue rectangle represents ORF1, grey rectangle represents a GAL4 tag, with expected molecular weights expressed in kilodaltons (kDa). Red asterisk denotes heterodimers. **B**. Western blot analysis of ORF1p species expressed by hORF1co (blue rectangle labeled hORF1co) and hGORF1co (grey/blue rectangle labeled hGORF1co) plasmids individually transfected (hORF1co lane and hGORF1co lane) or cotransfected (hORF1co+hGORF1co lane) in HeLa cells. Western blot analysis of ORF1 protein species in HeLa cells under non-reducing (left panel) and reducing (right panel) conditions. Proteins are detected with human specific polyclonal antibodies. GAPDH used as loading control. A molecular weight ladder in kilodaltons (kDa) is presented on right side. Red asterisk indicates an ORF1p heterodimer. **C**. The same experiment as in B is performed in NIH-3T3 cells. Non-reducing western blot conditions are shown. Red asterisk denotes ORF1p heterodimers. **D**. Quantitation of western blot results from B and C using heterodimer/ORF1p monomer ratio. Statistical significance is indicated by dashed lines. An asterisk indicates statistically significant difference (T-test, p-value<0.05).

It is known that protein-protein interactions can be stabilized by disulfide bonds. It has been previously demonstrated that ORF1p trimerization can be stabilized by disulfide bonds *in vitro* [19,20]. Traditional reducing SDS-PAGE protein fractionation eliminates these bonds, subsequently leading to the detection of only ORF1p monomers. However, under non-reducing fractionation conditions, these covalent interactions are preserved. Western blot analysis following non-reducing SDS-PAGE of hORF1co and hGORF1co individually transfected in HeLa cells readily detected different species of ORF1p including monomers, homodimers, and homotrimers (Figure 5B). Homodimer size is specific to each protein because of the difference in the size of monomers (hGORF1co, 57 kDa, *versus* hORF1co, 40 kDa). If these proteins can form a heterodimer by interacting *in trans*, a unique band is expected to be detected in cells cotransfected with both expression plasmids (Figure 5A, middle lane). Indeed, coexpression of the tagged and untagged ORF1 proteins in HeLa and NIH-3T3 cells produced the predicted band of molecular weight consistent with a hORF1co/hGORF1co heterodimer (Figure 5B and C, asterisk). Figure 5D demonstrates that human cells support a more efficient ORF1p interaction *in trans* than do mouse cells, providing a plausible explanation for the difference in the ORF1p self-interaction detected by the M2H system (Figure 1B). ORF1p *trans* interaction is not unique to the protein produced by the codon-optimized expression plasmid, as coexpression of hORF1wt and hGORF1co proteins also leads to heterodimer formation in HeLa cells. The detection of this heterodimer using both human ORF1p- and T7-specific antibodies (hORF1wt contains a unique T7 tag) demonstrates the presence of the hORF1wt protein in this unique band (Figure S5A and B). Together these data demonstrate that ORF1 proteins produced from different mRNAs can interact *in trans*.

The disulfide bonds that enable the above observations could have originated either in the cell or during cell lysis [33-35]. To determine the origin of disulfide bonds between ORF1 proteins, total cell lysates were collected in the presence of N-ethylmaleimide (NEM), which prevents *de novo* disulfide bond formation. Western blot analysis following non-reducing SDS-PAGE separation of these samples demonstrated a significant reduction of observable ORF1p dimers and trimers (Figure S6A), supporting that the majority of the disulfide bonds between ORF1p monomers occur during protein harvesting. Despite this, we contend that disulfide bond formation during lysis and harvest allows the capture of ORF1p self-interactions which occur within mammalian cells, as these bonds can only form between closely interacting proteins [33] (as also demonstrated by the following control experiments). 

We wished to further test whether the disulfide bonds between the ORF1p monomers could be formed either during cell lysis or sample processing. We performed a control experiment by comparing HeLa cells either cotransfected with GAL4-fused or non-fused ORF1 expression vectors, or separately transfected with those vectors and subsequently mixed together (Figure S6B). ORF1p heterodimerization may not be unique to the mammalian environment and the disulfide bonds may be formed during sample processing as opposed to during lysis of the cell. If either one of these possibilities is true, then the heterodimer band is expected to be detected by western blot analysis on the samples harvested from cells separately transfected with each expression vector and mixed prior to protein harvesting. Figure S6C demonstrates that ORF1p heterodimerization is only detected when ORF1 proteins are coexpressed in mammalian cells, as no ORF1p heterodimerization was observed when cells separately expressing GAL4-fused or non-fused ORF1 proteins were combined prior to sample processing. 

Furthermore, we investigated whether heterotrimer formation was possible outside the context of the intact cell as a function of time elapsed during sample processing. Protein samples harvested from cells transfected with either hORF1 or hGORF1 expression plasmid were mixed and incubated on ice in the presence or absence of NEM (Figure S7A). Western blot analysis of these mixed samples demonstrated that the ORF1p heterodimers cannot form *in vitro* under the conditions tested, as no unique bands (predicted as a result of said heterodimer formation) were observed (Figure S7B). Sonication of protein samples in the presence or absence of NEM also had no effect on ORF1p heterodimer formation (Figure S8A and B). 

Combined, these results demonstrate that heterodimer formation by the ORF1 proteins produced by the M2H expression plasmids in mammalian cells is a definite source of the signal detected by the M2H system. However, it may not be the only source of the signal because we cannot rule out the possibility that interactions between homotrimers could potentially contribute to the signal. Importantly, ORF1p monomers can efficiently self-interact *in trans*. Quantitative analysis of heterodimerization efficiency between hGORF1co and hORF1co in HeLa and NIH-3T3 cells demonstrated that human cells supported ORF1p *trans* interaction more efficiently than did mouse cells (Figure 5D), providing a reasonable explanation for the difference in the ORF1p self-interaction between these cells as detected by the M2H system (Figure 1). 

## Discussion

LINE-1 is the only currently active autonomous non-LTR retroelement in the human genome. It is responsible for the retrotransposition of itself and its parasites, the human SINE Alu and SVA elements [1-3]. The L1 replication cycle begins with the transcription of a functional L1 locus and ends with an L1 copy integrated in a new genomic location. The necessary replication cycle steps include L1 mRNA gaining access to the cytoplasm, where translation of the two L1 encoded proteins (ORF1p and ORF2p) takes place, followed by assembly of the functional L1 RNPs composed of the L1 mRNA andL1-encoded proteins [36]. These interactions exhibit a very strong *cis*-preference in mammalian cells [16]. In the context of L1 retrotransposition, *cis*-preference is understood to be the propensity of the L1 translated proteins to associate with their encoding RNA (constituting the L1 RNP), as opposed to other RNAs (either cellular or generated by other L1 loci). Thus, according to this model, there is expected there is a preference for trimerization between ORF1 proteins made from the same mRNA. Through a yet unknown mechanism, the RNPs return to the nucleus and interact with genomic DNA to initiate integration through target primed reverse transcription [37,38], a process which most likely depends on the many reported properties of the ORF1p [17]. 

Studies using *in vitro* systems have generated many important discoveries about human and mouse ORF1p self-interaction [18-20]. The CCD motif of the mouse ORF1p was identified to be a requirement for trimerization [14]. Despite the deletion of 7 out of 14 heptad repeats constituting the CCD, this observation was later confirmed for the human ORF1p when its crystal structure was solved [21]. A single cysteine present within the mouse ORF1p coiled-coiled domain was reported to be involved in the mouse ORF1p trimerization [19]. The human L1 ORF1p contains four cysteine residues, whose involvement in the trimerization process is not yet established, as most of the coiled-coil domain of the crystallized human ORF1p was deleted. 

Many (if not all) steps of the L1 replication cycle are downregulated by the host [36], most likely in an attempt by the host to mitigate the potential for genomic instability that can arise from an overly enthusiastic retroelement [29,39]. Thus, understanding ORF1p self-interaction in the mammalian environment is important for identifying any potential cellular mechanisms controlling ORF1p assembly. We utilized a mammalian two-hybrid system that captures the ability of both human and mouse ORF1 proteins to self-interact *in trans* in cultured mammalian cells (Figure 1). 

This system has many benefits for future L1 ORF1p studies, which are synergistically amplified when applied in combination with more traditional *in vitro* techniques. Among these advantages is the ease of the approach and detection of ORF1p self-interactions in the cellular environment. As the approach relies on transient transfections, the ORF1p self-interactions can be easily and rapidly tested in multiple cell types. The system does not require high protein expression levels, as both the codon-optimized and wild type mouse ORF1p produced a similar signal (Figure 1C). Furthermore, the effects of various treatments (particularly those affecting retrotransposition without altering L1 expression) on L1 ORF1p self-interaction could be conducted in different cell types. Finally, the system could also be used for detection and mechanistic studies of ORF1p interactions with specific cellular proteins or for testing the effects of overexpression or depletion of specific cellular factors on L1 ORF1p interaction. Additionally, a positive readout in the M2H system mimics steps of the L1 replication cycle related to ORF1p biology. 

As is the case with any experimental system, the M2H has its limitations. One of the main limitations of the system is that it cannot distinguish which ORF1p trans-interactions produce the positive signal, as *trans* interactions between monomers or homotrimers could contribute to the signal. Thus, the M2H assay cannot replace traditional *in vitro* approaches that clearly distinguish between these types of interactions. Furthermore, some wild type sequences, such as the wt human ORF1, may not produce any protein when subcloned into the M2H expression vectors, possibly due to the presence of functional splice sites [13,31,32] (Figure S3A) 

Characterization of ORF1p self-interactions using the M2H system resulted in a number of discoveries concerning ORF1p self-interaction. Although it has been previously reported that L1 proteins exhibit a strong *cis*-preference [16] (presuming that L1 ORF1p trimers are formed by the proteins translated from the same mRNA), we detected robust levels of ORF1p interaction between ORF1 proteins, which were produced from separate ORF1 expression plasmids (Figure 5 and Figure S5). While these results may initially seem to contradict previous observations, we think that they expand our understanding of ORF1p biology without arguing against *cis*-preference. While the significance of *cis*-preference in the L1 replication cycle is well understood, the effects of ORF1p *trans* interactions remain almost completely unexplored in respect to their significance to the L1 amplification. Indeed, it has already been reported that ORF1p enhances Alu retrotransposition [29], which is in itself reliant upon a type of *trans* interaction. 

ORF1p *trans* interaction may have important biological implications for the L1 replication cycle. There are many more L1 loci in the human and rodent genomes with non-functional ORF1p than those that are competent for retrotransposition [40]. Additionally, some prematurely polyadenylated and spliced L1 mRNAs [12,13] have the potential to produce functional and non-functional ORF1 proteins. Many of these defective loci are expressed in human cells, possibly providing a pool of non-functional ORF1p that may interact *in trans* with functional ORF1p most likely creating non-functional trimers with an as-yet unknown effect on retrotransposition of functional L1 elements.

The non-reducing western blot analysis of the ORF1p self-interaction demonstrated that disulfide bonds formed during cell lysis stabilize ORF1p interactions preexisting within mammalian cells (Figure 5 and Figures S5-S8), potentially providing a glimpse into the coordination of ORF1 monomers within the trimeric structure. It is known that these cysteine residues must be in extremely close proximity to one another for such bonds to form. Thus, the formation of these bonds is indicative of the very tight association that occurs between ORF1p monomers within the cellular context. Equally important is the observation that heterodimers with resultant disulfide bonds cannot be formed by the simple mixing of cellular lysates during harvest, demonstrating the importance of the cellular context to heterodimer formation.

While characterizing ORF1p self-interaction in the M2H system, we also observed that a substantial proportion of hORF1p and mORF1p localize to the nucleus in both HeLa and NIH-3T3 cells (Figures 3, 4 and Figures S3B, S3C, and S4). This pattern of subcellular localization was detected with GAL4-tagged ORF1 proteins expressed from codon-optimized vectors, and with the ORF1p generated from both wild type and codon-optimized ORF1 plasmids, and most importantly from wild type full-length L1 expression plasmids(Figure 4A and 4B). The dose curve experiment (Figure S4D), along with the comparisons of ORF1p expression between codon-optimized and wild type expression plasmids (Figure 4), demonstrate that the predominantly nuclear localization of ORF1p is not concentration dependent within the parameters of our experiments. The time course experiment confirmed that the abundant nuclear presence of ORF1p is not time dependent (Figures S4C and S4D). These findings are consistent with reports of nuclear detection of the endogenously expressed ORF1p in human tumor samples [25,26] and the recently reported ORF1p interaction profile with nuclear and cytoplasmic proteins [27]. 

There are several technical differences between the previous studies detecting L1 ORF1p predominantly in the cytoplasm and those detecting it in both cellular compartments. Several previous publications detected L1 ORF1p in the cytoplasm of cultured cells using IHC [24-26]. The experimental approach reported here relies on western blot analysis, which has very different sensitivity limits compared to IHC when detecting a potentially diffuse signal. We also utilized untagged ORF1p, cognizant of the fact that protein tags are notorious for their ability to alter mammalian protein function and/or subcellular localization [41]. Consistent with these observations, fusion of the VP16 tag to the N-terminus of human or mouse ORF1p significantly increased their cytoplasmic localization, despite the fact that a known NLS sequence is contained within the N-terminal VP16 fusion domain (Figures 1 and 3). This change in the subcellular localization is accompanied by reduced steady-state levels of the human and mouse VP16 ORF1 fusion proteins (Figure 3). While it was previously noted that some N-terminal fusions of ORF1p are not compatible with L1 retrotransposition [24], not every N-terminal fusion affects L1 ORF1p localization (Figure 3), suggesting that the altered subcellular localization is tag specific. 

One of the other technical aspects of the two approaches (western blot and IHC) to consider is the sensitivity and specificity of the antibody. We cannot rule out the possibility that our antibodies detect nuclear and cytoplasmic forms of ORF1p, whether fused or unfused, with different efficiencies due to potential post-translational modifications. The same consideration applies to the IHC approach, where natively folded proteins are expected to be detected with antibodies recognizing denatured proteins. Additionally, ORF1p epitopes may also be obscured by protein-protein interactions, which are preserved when using IHC. This is especially concerning given that the L1 RNP (including all cellular factors) is largely poorly understood, with many potential ORF1p interacting partners having been recently identified [27]. It is quite likely that ORF1p epitopes in the functional RNP may be obscured by attendant proteins preventing detection by the antibody. Our observations provide important experimental evidence that studies of tagged L1 proteins should be thoroughly validated, and comparisons of results obtained using different techniques should be interpreted with the consideration of the limitations associated with each approach. 

Since the exact mechanism responsible for nuclear L1 ORF1p localization remains unknown, our findings have important implications for the L1 replication cycle. It is currently not known how the L1 RNP gains access to the nucleus. It was previously proposed that ORF2p contains an NLS, which may facilitate nuclear entry of the L1 RNP [23]. Our data demonstrate that ORF1p may also contribute to L1 RNP entry into the nucleus, especially considering that it is likely that many more ORF1p than ORF2p molecules reside within an L1 RNP [20-22]. This role of L1 ORF1p may be extended to other L1-dependent retroelements that parasitize the L1 mobilization machinery. ORF1p is known to enhance Alu retrotransposition [29], and our data open the possibility that this improvement may be due to the ORF1p-assisted entry of Alu RNPs into the nucleus.

In summary, we report an assay that provides a relatively easy analysis of L1 ORF1p self-interaction in mammalian cells. Our study demonstrates that transiently expressed mouse and human ORF1 protein is readily detected in the nuclear fractions of mammalian cells of human and mouse origin. This finding suggests that the ORF1p may be aiding in L1 and Alu RNP access to the nucleus. We also show that L1 ORF1 protein can interact *in trans*, raising the possibility of L1 retrotransposition interference by L1 loci expressing non-functional ORF1 proteins.

## Materials and Methods

### Cells

NIH-3T3 (ATCC CRL-1658) and HeLa (ATCC CCL2) cells were maintained as previously described [12]. PC3 cells (ATCC CRL-1435) were cultured in HyClone Dulbecco’s modified Eagle’s medium (DMEM) with L-Glutamine and Sodium Pyruvate (Thermo Scientific) with 10% fetal bovine serum (Invitrogen) and maintained under 5% CO_2_ at 37^O^C. 

### Transfections

Western blot: HeLa and NIH-3T3 cells were seeded at 2x10^6^ and 2.5x10^6^ cells per T75 flask, respectively, and transfected 16-18 hours later with 5µg of expression plasmids containing wt and 3µg of plasmids containing codon-optimized mouse or human L1 sequences. Plus reagent (6µl) (Invitrogen) and lipofectamine (18µl (3T3) or 24µl (HeLa)) (Invitrogen) were used in the transfection reaction in serum-free media. Serum-free media was replaced with serum-containing media 3 hours after transfection and cells were harvested 24 hours later. Dose curve of ORF1p expression was performed by transfecting 1-6 micrograms of hORF1co expression plasmid, using the same conditions as above. Equal loading western blot: (Figure S4B). The loading amount of the western blot was the only deviation from the above mentioned western blot protocol. The amounts for each nuclear and cytoplasmic compartment pair were determined by the compartment that had the lowest percentage of total protein. The relative percentage of each compartment was between 2.1-3.8% of the total protein present in that compartment. Mammalian Two-hybrid (M2H, Promega): HeLa and 3T3 cells were seeded at 1.5x10^5^ and 1x10^5^ cells per well in a 6 well plate 18 hours prior to transfection. Each of the three M2H plasmids (0.1µg) were cotransfected using 1µl of plus reagent (Invitrogen) and 1.5µl of lipofectamine (Invitrogen). Media containing transfection cocktail was changed 3 hours after transfection and cells were harvested 48 hours later.

### Protein harvest for western blot analysis

Cells were harvested using Triton lysis buffer, TLB, (50 mM Tris, 150mM NaCl, 10 mM EDTA, 0.5% TritionX 0.5%, pH=7.2) for collection of cytoplasmic protein or Triton sodium dodecyl sulfate lysis buffer, TLB SDS, (50 mM Tris, 150mM NaCl, 10 mM EDTA, 0.5% sodium dodecyl sulfate, TritionX 0.5%, pH=7.2) for nuclear and total protein harvesting. Lysis buffers were supplemented with Halt Protease inhibitors, and phosphatase inhibitors 2 and 3 (Sigma) at 10µl/mL each. The total and nuclear lysates were sonicated three times for 10 seconds at 12 watts RMS using a 3mm wide QSonica Microson homogenizer with Microson ultrasonic cell disruptor XL2000 (Microson).The protein concentrations of the lysates were determined using 595nm wavelength OD values against a Bovine Serum Albumin (BSA) standard. 

For western blot analysis, samples (10-40µg) were combined with 2× Laemmli buffer, 1.6µl β-mercaptoethanol and boiled for 5 minutes prior to loading. Samples were fractionated on Bis-Tris 4-12% Midi gels (Invitrogen) and transferred to nitrocellulose membranes (iBlot System; Invitrogen). Membranes were blocked for 1 hour in 5% milk in PBS-Tween and incubated with hORF1 (custom polyclonal Rabbit antibodies, epitope: TGNSKTQSASPPPK), mORF1 (custom polyclonal Goat antibodies, epitope: YRTPNRLDQKRNSS) or T7 (Cell signaling 6885s) antibodies overnight at 4^O^C. Detection was carried out using horseradish peroxidase-conjugated secondary antibodies (Santa Cruz sc-2317 or Santa Cruz sc-2020) and developed using Immun-Star™ WesternC™ Kit (Bio-Rad, Cat. #170-5070). Equal loading and the quality of subcellular fractionation was confirmed with GAPDH antibodies (Santa Cruz sc-25778), Lamin A/C (Cell signaling 2032S) or Calregulin (Santa Cruz T-19) using the same protocol. 

Non-reducing gels (Figure 5 and Figures S5, S6, S7 and S8). Transfected cells were lysed using TLB buffer supplemented with 0, 1, 13, or 25mM of N-ethylmaleimide (NEM) (Sigma E3876) dissolved in 100% ethanol (Sigma E7023). Non-NEM samples were supplemented with 100% ethanol equal in volume to the added NEM. The samples were sonicated 5 times for 5 seconds and kept on ice between sonications. Samples were combined with 2× Laemmli buffer without β-mercaptoethanol and heated at 85^O^C for 5 minutes. Samples were fractionated on non-reducing denaturating SDS -PAGE (Bis-Tris 4-12% Midi gels (Invitrogen)) gels. Unless otherwise indicated, 25 mM of NEM was used. 

Heterodimer formation analysis HeLa cells were individually transfected with ORF1 expression plasmids. Cells were transiently transfected with hORF1 or hGORF1 expression plasmids, trypsinized 24 hours later and mixed in equal amounts (Figure S6). Total protein from the cell mixture was harvested as above with or without NEM. Protein samples were harvested without NEM from NIH-3T3 cells transiently transfected with hORF1 or hGORF1, then mixed in 1:1 ratio and incubated for 0, 5, 10, or 15 minutes (Figure S7). Total protein was harvested with or without NEM from NIH-3T3 cells cotransfected with hORF1 and hGORF1 expression plasmids. Cells were then exposed to additional sonication in the presence of absence of NEM (Figure S8). 

### Mammalian Two-Hybrid

Cells were harvested by scraping into 250 µL of 1x passive lysis buffer (Promega, E1960) subjected to freeze(-80^O^C)/thaw(25^O^C) lysis x3.  Supernatant was collected after centrifugation for 30 seconds at 1.61x10^4^rpm at 4^O^C. The protein was quantitated using a BSA assay as described above. The cellular lysates were assayed according to manufacturer's protocol (Promega E1960, E2440) using Sirius luminometer (Titertek Berthold, Pforzheim, Germany).

### Plasmids

(Table **S1**) “hL1wt” is JM101/L1.3 no tag (L1.3) [16]. “hORF1co” is pBudORF1opt [29]. “mORF1co” is pBudORF1_syn_ [30]. “mL1wt” is L1_Spa_ [42]. “hORF1wt” is pBudORF1 L1.3 [29]. Human and mouse wild type L1 sequences used for cloning are L1.2 (L19088) and L1spa (AF160099) elements. pBud and pBudCE4.1 (Invitrogen) were used as the empty vector controls. hGORF1co expression plasmid was generated by PCR amplification of “hORF1co” with primers containing SgfI and PmeI restriction sites and subcloning the PCR fragment into the pFN11A vector (Promega). hVORF1co was generated by PCR amplification of “hORF1co” with primers containing SgfI and PmeI restriction sites and subcloning the PCR fragment into the pFN10A vector (Promega). The same approach using the above-mentioned expression vectors was used to generate hGORF1wt, hVORF1wt, mGORF1co, mVORF1co, mGORF1wt, mVORF1wt expression plasmids.

### Statistical Analysis

Statistical significance was determined using a one-tailed T-test with equal variance in excel using N=3 to 5. Error bars represent standard deviation.

## Supporting Information

Figure S1 **Analysis of human and mouse ORF1p self-interaction using the M2H system.** M2H results for hVORF1co + mGORF1co and mVORF1co + hGORF1co interaction in HeLa cells normalized to positive control. No interaction is detected between these protein pairs (T-test, p-value >0.05).
(TIF)

Figure S2 **Validation of human- and mouse-specific ORF1p antibodies.** **A**. Human ORF1 polyclonal antibodies were generated against a peptide within the N-terminus of the hORF1p. Western blot analysis of total cellular lysates harvested from HeLa cells transfected with a plasmid expressing hORF1co protein (blue rectangle). (**Left to right**) First Panel: detection of hORF1p in HeLa cells (transiently transfected with an empty control vector, hORF1wt, hL1wt or hORF1co plasmids) with human ORF1p-specific antibodies; Second Panel: detection of hORF1p in HeLa cells with human ORF1p-specific antibodies and the addition of the peptide used for the antibodies’ generation (for the peptide sequence, see material and methods); Third Panel: detection of hORF1p in HeLa cells with secondary antibodies alone. **B**. Mouse ORF1 polyclonal antibodies were generated against a peptide located within the C-terminus of mORF1p (green rectangle). Western blot analysis of protein lysates from HeLa cells transiently transfected with mL1 wt and mORF1co expression plasmids. mORF1co expression plasmid adds a tag to the ORF1p, causing the difference in observed molecular weight between mL1wt and mORF1co. (**Left to right**) Detection of mORF1p expressed in cells transiently transfected with mL1wt and mORF1co expression plasmids. Loss of protein detection in the presence of mORF1 peptide (for peptide sequence see material and methods) pre-incubated with mORF1 antibodies prior to use for western blot analysis. The third group is incubated with secondary goat antibodies only.
(TIF)

Figure S3 **Analysis of subcellular localization of ORF1 fusion proteins expressed by plasmids containing wild type L1 sequence.** **A**. Western blot analysis of total hORFwt (blue rectangle), hGORF1wt or hVORF1wt (grey/blue rectangle) protein transiently expressed in HeLa cells. GAPDH is used as a loading control. Control lanes indicate cells transfected with an empty vector. Only untagged hORF1wt is detected. **B**. Western blot analysis of nuclear and cytoplasmic fractions of mORF1co (green rectangle), mGORF1wt and mVORF1wt (grey/green rectangle) proteins transiently expressed in HeLa cells. Proteins in nuclear (N) and cytoplasmic (C) fractions were detected with mouse specific ORF1p polyclonal antibodies. GAPDH (cytoplasmic marker) and Lamin A (nuclear marker) are used as loading and cell fractionation controls. 50 and 75 kDa indicate molecular weights. Control lanes indicate cells transfected with an empty vector. Red asterisk indicates the mORF1p-specific band detected in the nuclear and cytoplasmic fractions. **C**. The same experiment and analysis as in B is performed in NIH-3T3 cells. Red asterisk represents mORF1p specific band, black asterisk represents a non-specific band.
(TIF)

Figure S4 **Effect of cell type and transfection conditions on the subcellular localization of hORF1p.** **A**. Western blot analysis of hORF1co transiently expressed in a prostate cancer cell line (PC3). Protein is detected with human specific ORF1p polyclonal antibodies. GAPDH (cytoplasmic marker) is used as a loading and cell fractionation control. Control lanes indicate cells transfected with empty vector. **B**. Western blot analysis of hORF1co (blue rectangle), hGORF1co and hVORF1co (grey/blue rectangle), and hL1wt and hORF1wt transiently expressed in HeLa cells. Equal fractions of the total protein in nuclear and cytoplasmic extracts for each construct transiently transfected in HeLa cells were analyzed. This approach is used to take into account the difference in the total amount of protein collected from each cellular fraction. Compare these results with the results shown in Figure 4, where the analysis was done by loading the same amount of protein for each subcellular fraction. Proteins in nuclear (N) and cytoplasmic (C) fractions were detected with human ORF1p-specific polyclonal antibodies. GAPDH (cytoplasmic marker) and Lamin A (nuclear marker) are used as loading and cell fractionation controls. 50 and 75 kDa are molecular weights. Control lanes indicate cells transfected with empty vector. **C**. Time course analysis of hL1wt protein expressed in transiently transfected HeLa cells. Cells were harvested over a 48 hour period at indicated time points (h) post transfection. hORF1p in nuclear (N) and cytoplasmic (C) fractions was detected with human specific ORF1p polyclonal antibodies. GAPDH (cytoplasmic marker) and Lamin A (nuclear marker) are used as loading and cell fractionation controls. **D**. Western blot analysis of a dose curve: HeLa cells were transfected with increasing amounts of hORF1co plasmid (1 to 6 micrograms (µg)). The same loading controls and antibodies were used as in B and C. 37 kDa is a molecular weight. **E**. Multiple conditions for processing were used to rule out the effect of the duration of cell lysis and sample processing on ORF1p subcellular localization. The duration of cell lysis on ice, centrifugation time, or centrifugation speed did not have any effect on the ORF1p subcellular localization. ORF1p in nuclear (N) and cytoplasmic (C) fractions was detected with human-specific ORF1p polyclonal antibodies. GAPDH (cytoplasmic marker) and Lamin A (nuclear marker) are used as loading and cell fractionation controls. Control lanes indicate cells transfected with empty vector.
(TIF)

Figure S5 **ORF1p heterodimer formation analysis A.** Western blot analysis of ORF1p expressed by hORF1wt (blue rectangle labeled hORF1wt) and hGORF1co (grey/blue rectangle labeled hGORF1co) vectors transiently transfected alone or cotransfected in HeLa cells. Red asterisk denotes an ORF1p heterodimer. Proteins are detected with human specific polyclonal antibodies. GAPDH was used as loading control. Molecular weights are indicated on the right in kilodaltons (kDa). **B**. The same experiment as in A. T7 tag-specific antibodies were used for protein detection. Red asterisk denotes an ORF1p heterodimer. GAPDH was used as loading control. Molecular weights are indicated on the right in kilodaltons (kDa).
(TIF)

Figure S6 **Disulfide bond formation and ORF1p self-interaction.** **A**. Western blot analysis of hORF1co expressed in HeLa cells transiently transfected with hORF1co expression plasmid. Total protein was harvested in the presence of increasing amounts of N-ethylmaleimide (NEM) and analyzed on a non-reducing SDS-PAGE gel. Molecular weights in kilodaltons (kDa) are indicated on the right. **B**. Schematic of the experimental approach. Total protein was collected with or without NEM (25mM) from HeLa cells cotransfected with hORF1co and hGORF1co expression plasmids (see left, labeled cotransfected) or transfected individually (see right, labeled mixed). The control is designed to determine whether hORF1p and hGORF1p can interact during sample processing. Briefly, cells transfected with either hORF1 or hGORF1 expression plasmids were trypsinized and mixed in equal amounts. Total protein was extracted from the mixture with or without the addition of NEM. No interactions *in* *trans* were detected under these experimental conditions. **C**. Western blot of ORF1p (blue rectangle labeled hORF1co is unfused, grey/blue rectangle labeled hGORF1co is a fusion ORF1 protein) interactions *in* *trans* occurring within mammalian cells. Proteins are detected with human-specific ORF1p polyclonal antibodies. GAPDH used as loading control. Molecular weights in kilodaltons (kDa) are shown on the right. Red asterisk is an ORF1p heterodimer.
(TIF)

Figure S7 **Analysis of ORF1p species as a function of incubation time.** **A**. Schematic of experiment. Cells are transfected individually with either hORF1co or hGORF1co expression plasmids and processed separately. Samples are combined prior to loading on western blot for indicated co-incubation time (see B), with or without NEM (25mM). **B**. Western blot analysis of ORF1p species formed during protein harvesting detects monomer, dimers, and trimers. No heterodimer formation was observed under these experimental conditions. Proteins are detected with human-specific ORF1p polyclonal antibodies. GAPDH used as loading control. Molecular weights in kilodaltons (kDa) are displayed on the right.
(TIF)

Figure S8 **Sonication following protein harvesting does not effect observed ORF1p species.** **A**. Schematic of experiment. Cells are transfected individually or cotransfected with hORF1co and/or hGORF1co, and lysates are harvested without NEM. After processing, samples are split into two tubes, with one tube having 25 mM NEM added and the other with an equivalent amount of ethanol. Samples were then sonicated and fractionated on an SDS-PAGE gel under non-reducing conditions (see materials and methods). **B**. Western blot analysis of ORF1p species formed during protein harvesting detects heterodimers which are stable regardless of sonication or the presence/absence of NEM. Proteins are detected with human-specific ORF1p polyclonal antibodies. GAPDH used as loading control. Molecular weights in kilodaltons (kDa) are displayed on the right.
(TIF)

Table S1 **Constructs utilized in the study of ORF1p self-interaction.** Constructs expressing ORF1p of human and mouse origin are shown. Abbreviations are shown on the left with the parent construct given on the right.
(TIF)

## References

[1] Moran JV, Holmes SE, Naas TP, DeBerardinis RJ, Boeke JD et al. (1996) High Frequency Retrotransposition in Cultured Mammalian Cells. Cell 87: 917-927. doi:10.1016/S0092-8674(00)81998-4. PubMed: 8945518. 8945518

[2] Dewannieux M, Esnault C, Heidmann T (2003) LINE-mediated retrotransposition of marked Alu sequences. Nat Genet 35: 41-48. doi:10.1038/ng1223. PubMed: 12897783. 12897783

[3] Ostertag EM, Goodier JL, Zhang Y, Kazazian HH Jr (2003) SVA Elements Are Nonautonomous Retrotransposons that Cause Disease in Humans. American Journal of Human Genetics 73: 1444-1451. doi:10.1086/380207. 14628287 PMC1180407

[4] (2001) Initial sequencing and analysis of the human genome. Nature 409: 860-921. doi:10.1038/35057062. PubMed: 11237011. 11237011

[5] Konkel MK, Wang J, Liang P, Batzer MA (2007) Identification and characterization of novel polymorphic LINE-1 insertions through comparison of two human genome sequence assemblies. Gene 390: 28-38. doi:10.1016/j.gene.2006.07.040. PubMed: 17034961. 17034961

[6] Beck CR, Collier P, Macfarlane C, Malig M, Kidd JM et al. (2010) LINE-1 Retrotransposition Activity in Human Genomes. Cell 141: 1159-1170. doi:10.1016/j.cell.2010.05.021. PubMed: 20602998. 20602998 PMC3013285

[7] Huang CRL, Schneider AM, Lu Y, Niranjan T, Shen P et al. (2010) Mobile Interspersed Repeats Are Major Structural Variants in the Human Genome. Cell 141: 1171-1182. doi:10.1016/j.cell.2010.05.026. PubMed: 20602999. 20602999 PMC2943426

[8] Ewing AD, Kazazian HH (2011) Whole-genome resequencing allows detection of many rare LINE-1 insertion alleles in humans. Genome Research 21: 985-990. doi:10.1101/gr.114777.110. PubMed: 20980553. 20980553 PMC3106331

[9] Kazazian HH, Wong C, Youssoufian H, Scott AF, Phillips DG et al. (1988) Haemophilia A resulting from de novo insertion of L1 sequences represents a novel mechanism for mutation in man. Nature 332: 164-166. doi:10.1038/332164a0. PubMed: 2831458. 2831458

[10] Woods-Samuels P, Wong C, Mathias SL, Scott AF, Kazazian HH Jr et al. (1989) Characterization of a nondeleterious L1 insertion in an intron of the human factor VIII gene and further evidence of open reading frames in functional L1 elements. Genomics 4: 290-296. doi:10.1016/0888-7543(89)90332-7. PubMed: 2497061. 2497061

[11] Alisch RS, Garcia-Perez JL, Muotri AR, Gage FH, Moran JV (2006) Unconventional translation of mammalian LINE-1 retrotransposons. Genes and Development 20: 210-224. doi:10.1101/gad.1380406. 16418485 PMC1356112

[12] Perepelitsa-Belancio V, Deininger P (2003) RNA truncation by premature polyadenylation attenuates human mobile element activity. Nat Genet 35: 363-366. doi:10.1038/ng1269. PubMed: 14625551. 14625551

[13] Belancio VP, Hedges DJ, Deininger P (2006) LINE-1 RNA splicing and influences on mammalian gene expression. Nucleic Acids Res 34: 1512-1521. doi:10.1093/nar/gkl027. PubMed: 16554555. 16554555 PMC1415225

[14] Kolosha VO, Martin SL (1997) In vitro properties of the first ORF protein from mouse LINE-1 support its role in ribonucleoprotein particle formation during retrotransposition. Proceedings of the National Academy of Sciences of the USA 94: 10155-10160. doi:10.1073/pnas.94.19.10155. 9294179 PMC23331

[15] Kulpa DA, Moran JV (2005) Ribonucleoprotein particle formation is necessary but not sufficient for LINE-1 retrotransposition. Hum Mol Genet 14: 3237-3248. doi:10.1093/hmg/ddi354. PubMed: 16183655. 16183655

[16] Wei W, Gilbert N, Ooi SL, Lawler JF, Ostertag EM et al. (2001) Human L1 Retrotransposition: cisPreference versus trans Complementation. Mol Cell Biol 21: 1429-1439. doi:10.1128/MCB.21.4.1429-1439.2001. PubMed: 11158327. 11158327 PMC99594

[17] Martin SL (2010) Nucleic acid chaperone properties of ORF1p from the non-LTR retrotransposon, LINE-1. RNA Biol 7: 706-711. doi:10.4161/rna.7.6.13766. PubMed: 21045547. 21045547 PMC3073329

[18] Martin SL, Bushman FD (2001) Nucleic Acid Chaperone Activity of the ORF1 Protein from the Mouse LINE-1 Retrotransposon. Mol Cell Biol 21: 467-475. doi:10.1128/MCB.21.2.467-475.2001. PubMed: 11134335. 11134335 PMC86601

[19] Martin SL, Branciforte D, Keller D, Bain DL (2003) Trimeric structure for an essential protein in L1 retrotransposition. Proc Natl Acad Sci U S A 100: 13815-13820. doi:10.1073/pnas.2336221100. PubMed: 14615577. 14615577 PMC283504

[20] Callahan KE, Hickman AB, Jones CE, Ghirlando R, Furano AV (2012) Polymerization and nucleic acid-binding properties of human L1 ORF1 protein. Nucleic Acids Research 40: 813-827. doi:10.1093/nar/gkr728. PubMed: 21937507. 21937507 PMC3258132

[21] Khazina E, Truffault V, Büttner R, Schmidt S, Coles M et al. (2011) Trimeric structure and flexibility of the L1ORF1 protein in human L1 retrotransposition. Nat Struct Mol Biol 18: 1006-1014. doi:10.1038/nsmb.2097. PubMed: 21822284. 21822284

[22] Basame S, Wai-lun Li P, Howard G, Branciforte D, Keller D, et al. (2006) Spatial Assembly and RNA Binding Stoichiometry of a LINE-1 Protein Essential for Retrotransposition. Journal of Molecular Biology 357: 351-357 10.1016/j.jmb.2005.12.063 16434051

[23] Goodier JL, Ostertag EM, Engleka KA, Seleme MC, Kazazian HH (2004) A potential role for the nucleolus in L1 retrotransposition. Hum Mol Genet 13: 1041-1048. doi:10.1093/hmg/ddh118. PubMed: 15028673. 15028673

[24] Goodier JL, Zhang L, Vetter MR, Kazazian HH (2007) LINE-1 ORF1 Protein Localizes in Stress Granules with Other RNA-Binding Proteins, Including Components of RNA Interference RNA-Induced Silencing Complex. Mol Cell Biol 27: 6469-6483. doi:10.1128/MCB.00332-07. PubMed: 17562864. 17562864 PMC2099616

[25] Harris CR, Normart R, Yang Q, Stevenson E, Haffty BG et al. (2010) Association of Nuclear Localization of a Long Interspersed Nuclear Element-1 Protein in Breast Tumors with Poor Prognostic Outcomes. Genes Cancer 1: 115-124. PubMed: 20948976. 20948976 10.1177/1947601909360812 PMC2952938

[26] Chen L, Dahlstrom JE, Chandra A, Board P, Rangasamy D (2012) Prognostic value of LINE-1 retrotransposon expression and its subcellular localization in breast cancer. Breast Cancer Res Treat 136: 129-142. doi:10.1007/s10549-012-2246-7. PubMed: 23053642. 23053642 PMC3473189

[27] Goodier JL, Cheung LE, Kazazian HH (2013) Mapping the LINE1 ORF1 protein interactome reveals associated inhibitors of human retrotransposition. Nucleic Acids Research. 10.1093/nar/gkt512 PMC3753637 23749060

[28] Goodier JL, Cheung LE, Kazazian HH Jr. (2012) MOV10 RNA Helicase Is a Potent Inhibitor of Retrotransposition in Cells. PLOS Genet 8: e1002941. 23093941 10.1371/journal.pgen.1002941 PMC3475670

[29] Wallace N, Wagstaff BJ, Deininger PL, Roy-Engel AM (2008) LINE-1 ORF1 protein enhances Alu SINE retrotransposition. Gene 419: 1-6. doi:10.1016/j.gene.2008.04.007. PubMed: 18534786. 18534786 PMC2491492

[30] Han JS, Boeke JD (2004) A highly active synthetic mammalian retrotransposon. Nature 429: 314-318. doi:10.1038/nature02535. PubMed: 15152256. 15152256

[31] Belancio VP (2011) Importance of RNA analysis in interpretation of reporter gene expression data. Analytical Biochemistry 417: 159-161. doi:10.1016/j.ab.2011.05.035. PubMed: 21693100. 21693100 PMC3145209

[32] Dmitriev SE, Andreev DE, Terenin IM, Olovnikov IA, Prassolov VS et al. (2007) Efficient Translation Initiation Directed by the 900-Nucleotide-Long and GC-Rich 5′ Untranslated Region of the Human Retrotransposon LINE-1 mRNA Is Strictly Cap Dependent Rather than Internal Ribosome Entry Site Mediated. Molecular and Cellular Biology 27: 4685-4697. doi:10.1128/MCB.02138-06. PubMed: 17470553. 17470553 PMC1951496

[33] Fass D (2012) Disulfide Bonding in Protein Biophysics. Annu Rev Biophys 41: 63-79. doi:10.1146/annurev-biophys-050511-102321. PubMed: 22224600. 22224600

[34] Dick TP, Bangia N, Peaper DR, Cresswell P (2002) Disulfide Bond Isomerization and the Assembly of MHC Class I-Peptide Complexes. Immunity 16: 87-98. doi:10.1016/S1074-7613(02)00263-7. PubMed: 11825568. 11825568

[35] Molinari M, Helenius A (1999) Glycoproteins form mixed disulphides with oxidoreductases during folding in living cells. Nature 402: 90-93. doi:10.1038/47062. PubMed: 10573423. 10573423

[36] Belancio VP, Hedges DJ, Deininger P (2008) Mammalian non-LTR retrotransposons: For better or worse, in sickness and in health. Genome Res 18: 343-358. doi:10.1101/gr.5558208. PubMed: 18256243. 18256243

[37] Luan DD, Eickbush TH (1996) Downstream 28S gene sequences on the RNA template affect the choice of primer and the accuracy of initiation by the R2 reverse transcriptase. Molecular and Cellular Biology 16: 4726-4734. PubMed: 8756630. 8756630 10.1128/mcb.16.9.4726 PMC231473

[38] Kulpa DA, Moran JV (2006) Cis-preferential LINE-1 reverse transcriptase activity in ribonucleoprotein particles. Nat Struct Mol Biol 13: 655-660. doi:10.1038/nsmb1107. PubMed: 16783376. 16783376

[39] Gasior SL, Preston G, Hedges DJ, Gilbert N, Moran JV et al. (2007) Characterization of pre-insertion loci of de novo L1 insertions. Gene 390: 190-198. doi:10.1016/j.gene.2006.08.024. PubMed: 17067767. 17067767 PMC1850991

[40] Penzkofer T, Dandekar T, Zemojtel T (2005) L1Base: from functional annotation to prediction of active LINE-1 elements. Nucleic Acids Res 33: D498-D500. PubMed: 15608246. 15608246 10.1093/nar/gki044 PMC539998

[41] Tavare JM, Fletcher LM, Welsh GI (2001) Using green fluorescent protein to study intracellular signalling. Journal of Endocrinology 170: 297-306. doi:10.1677/joe.0.1700297. PubMed: 11479127. 11479127

[42] Naas TP, DeBerardinis RJ, Moran JV, Ostertag EM, Kingsmore SF et al. (1998) An actively retrotransposing, novel subfamily of mouse L1 elements. EMBO J 17: 590-597. doi:10.1093/emboj/17.2.590. PubMed: 9430649. 9430649 PMC1170408

