# Supplementary figures and images for: Characterization of L1 ORF1p Self-Interaction and Cellular Localization Using a Mammalian Two-Hybrid System
Source: PLoS One. 2013 Dec 4;8(12):e82021. doi: 10.1371/journal.pone.0082021 (PMC3852968; doi:10.1371/journal.pone.0082021)

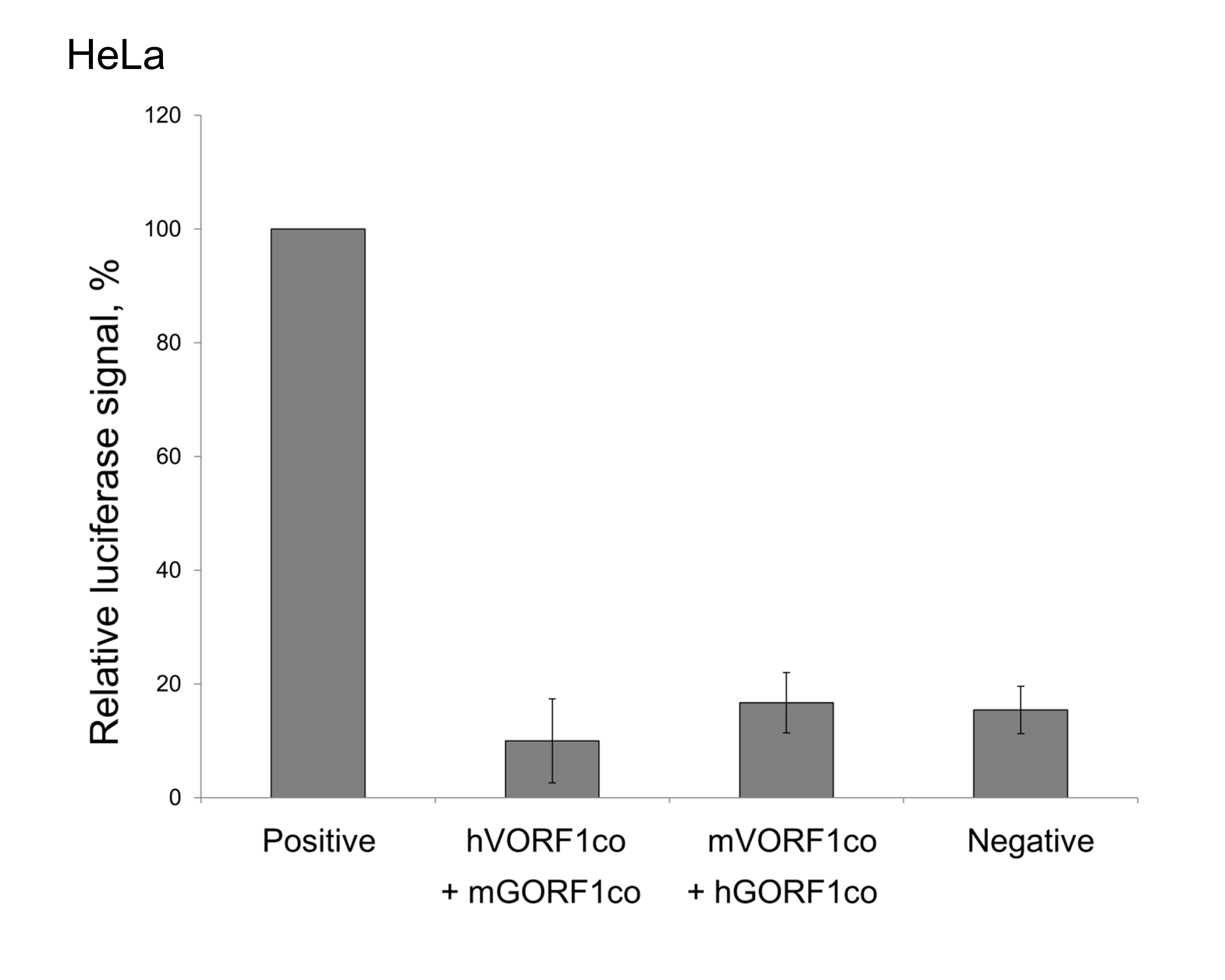

Supplement: Figure S1 — Analysis of human and mouse ORF1p self-interaction using the M2H system. M2H results for hVORF1co + mGORF1co and mVORF1co + hGORF1co interaction in HeLa cells normalized to positive control. No interaction is detected between these protein pairs (T-test, p-value >0.05). (TIF) [file pone.0082021.s001.tif]

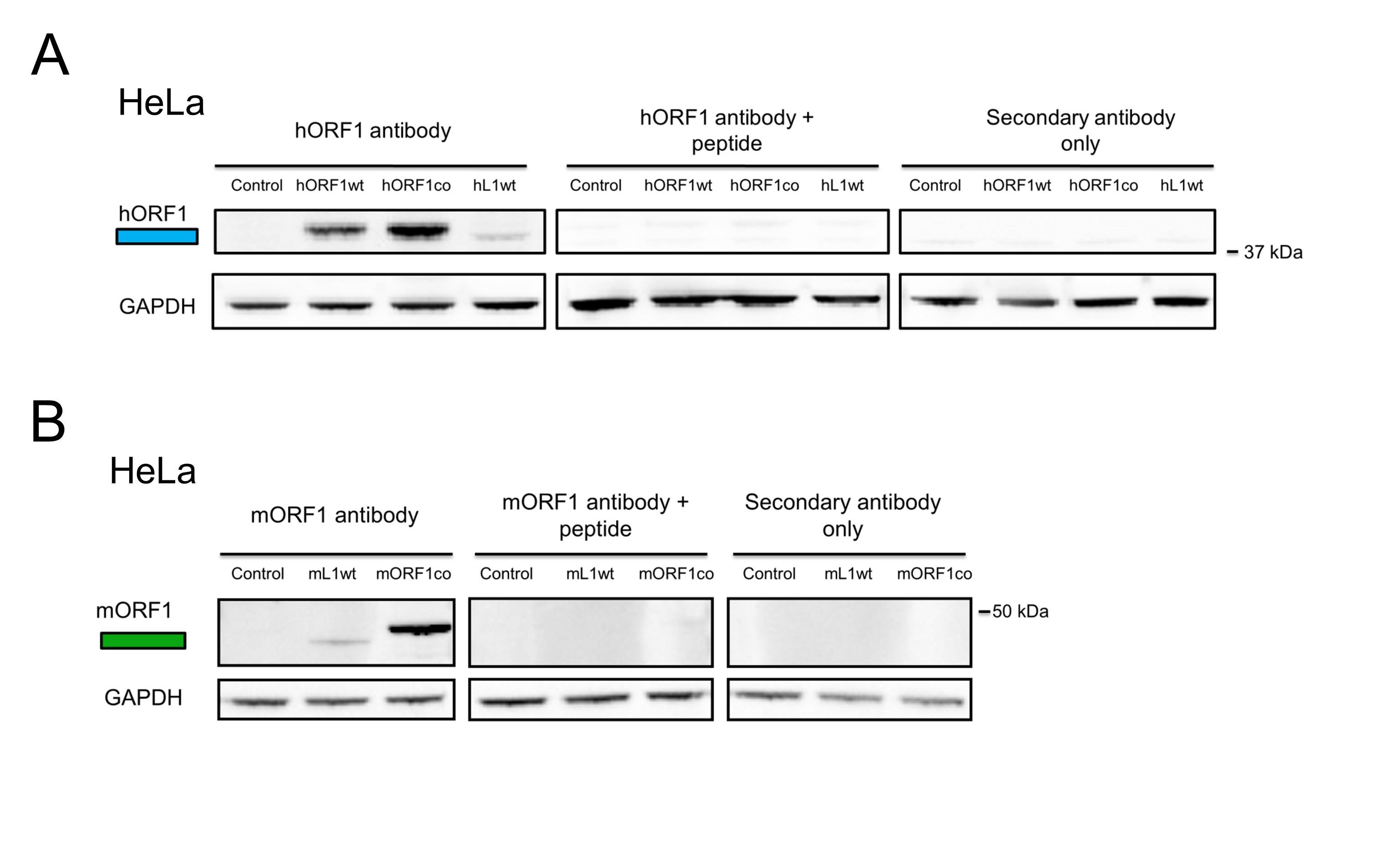

Supplement: Figure S2 — Validation of human- and mouse-specific ORF1p antibodies. A. Human ORF1 polyclonal antibodies were generated against a peptide within the N-terminus of the hORF1p. Western blot analysis of total cellular lysates harvested from HeLa cells transfected with a plasmid expressing hORF1co protein (blue rectangle). (Left to right) First Panel: detection of hORF1p in HeLa cells (transiently transfected with an empty control vector, hORF1wt, hL1wt or hORF1co plasmids) with human ORF1p-specific antibodies; Second Panel: detection of hORF1p in HeLa cells with human ORF1p-specific antibodies and the addition of the peptide used for the antibodies’ generation (for the peptide sequence, see material and methods); Third Panel: detection of hORF1p in HeLa cells with secondary antibodies alone. B. Mouse ORF1 polyclonal antibodies were generated against a peptide located within the C-terminus of mORF1p (green rectangle). Western blot analysis of protein lysates from HeLa cells transiently transfected with mL1 wt and mORF1co expression plasmids. mORF1co expression plasmid adds a tag to the ORF1p, causing the difference in observed molecular weight between mL1wt and mORF1co. (Left to right) Detection of mORF1p expressed in cells transiently transfected with mL1wt and mORF1co expression plasmids. Loss of protein detection in the presence of mORF1 peptide (for peptide sequence see material and methods) pre-incubated with mORF1 antibodies prior to use for western blot analysis. The third group is incubated with secondary goat antibodies only. (TIF) [file pone.0082021.s002.tif]

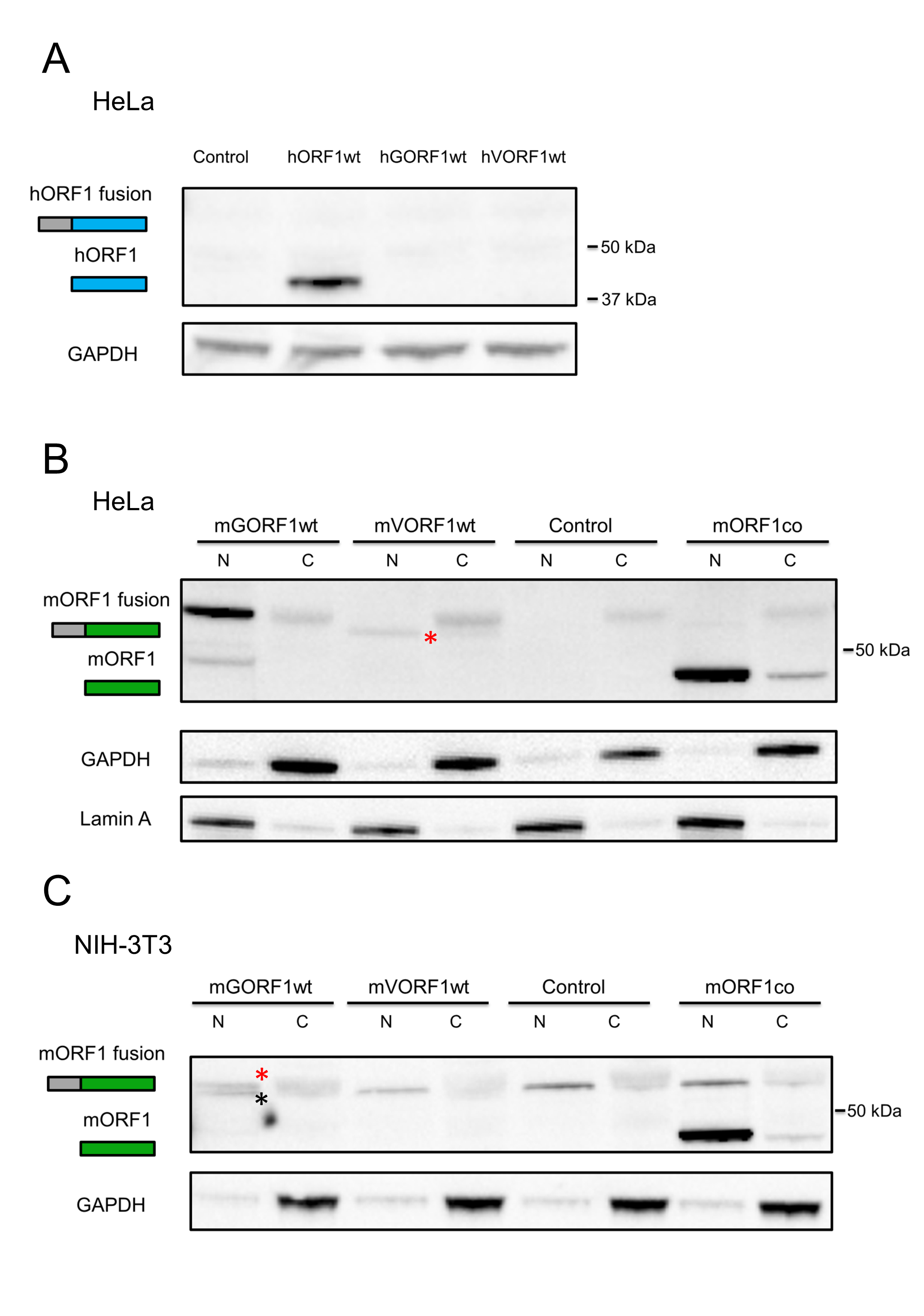

Supplement: Figure S3 — Analysis of subcellular localization of ORF1 fusion proteins expressed by plasmids containing wild type L1 sequence. A. Western blot analysis of total hORFwt (blue rectangle), hGORF1wt or hVORF1wt (grey/blue rectangle) protein transiently expressed in HeLa cells. GAPDH is used as a loading control. Control lanes indicate cells transfected with an empty vector. Only untagged hORF1wt is detected. B. Western blot analysis of nuclear and cytoplasmic fractions of mORF1co (green rectangle), mGORF1wt and mVORF1wt (grey/green rectangle) proteins transiently expressed in HeLa cells. Proteins in nuclear (N) and cytoplasmic (C) fractions were detected with mouse specific ORF1p polyclonal antibodies. GAPDH (cytoplasmic marker) and Lamin A (nuclear marker) are used as loading and cell fractionation controls. 50 and 75 kDa indicate molecular weights. Control lanes indicate cells transfected with an empty vector. Red asterisk indicates the mORF1p-specific band detected in the nuclear and cytoplasmic fractions. C. The same experiment and analysis as in B is performed in NIH-3T3 cells. Red asterisk represents mORF1p specific band, black asterisk represents a non-specific band. (TIF) [file pone.0082021.s003.tif]

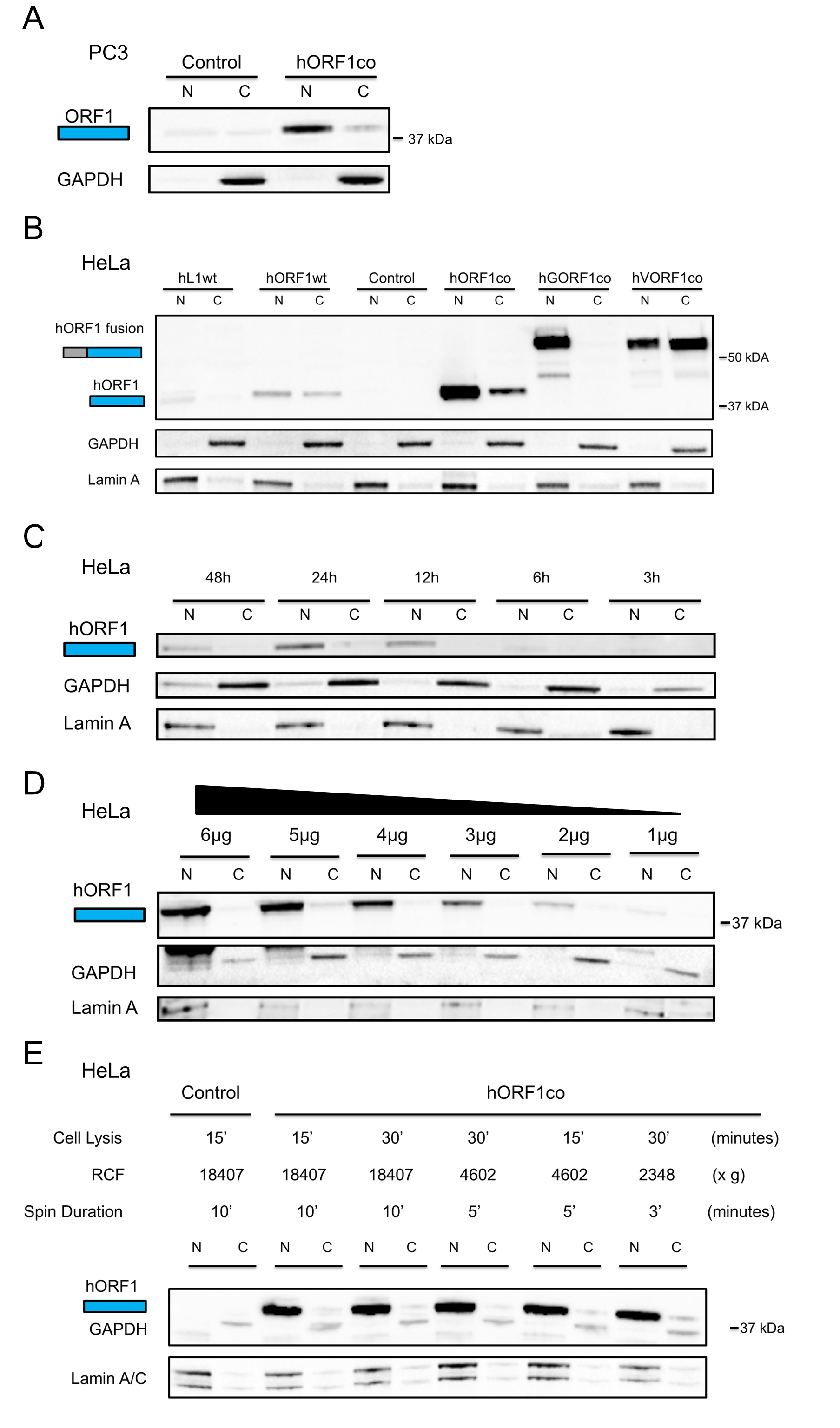

Supplement: Figure S4 — Effect of cell type and transfection conditions on the subcellular localization of hORF1p. A. Western blot analysis of hORF1co transiently expressed in a prostate cancer cell line (PC3). Protein is detected with human specific ORF1p polyclonal antibodies. GAPDH (cytoplasmic marker) is used as a loading and cell fractionation control. Control lanes indicate cells transfected with empty vector. B. Western blot analysis of hORF1co (blue rectangle), hGORF1co and hVORF1co (grey/blue rectangle), and hL1wt and hORF1wt transiently expressed in HeLa cells. Equal fractions of the total protein in nuclear and cytoplasmic extracts for each construct transiently transfected in HeLa cells were analyzed. This approach is used to take into account the difference in the total amount of protein collected from each cellular fraction. Compare these results with the results shown in Figure 4, where the analysis was done by loading the same amount of protein for each subcellular fraction. Proteins in nuclear (N) and cytoplasmic (C) fractions were detected with human ORF1p-specific polyclonal antibodies. GAPDH (cytoplasmic marker) and Lamin A (nuclear marker) are used as loading and cell fractionation controls. 50 and 75 kDa are molecular weights. Control lanes indicate cells transfected with empty vector. C. Time course analysis of hL1wt protein expressed in transiently transfected HeLa cells. Cells were harvested over a 48 hour period at indicated time points (h) post transfection. hORF1p in nuclear (N) and cytoplasmic (C) fractions was detected with human specific ORF1p polyclonal antibodies. GAPDH (cytoplasmic marker) and Lamin A (nuclear marker) are used as loading and cell fractionation controls. D. Western blot analysis of a dose curve: HeLa cells were transfected with increasing amounts of hORF1co plasmid (1 to 6 micrograms (µg)). The same loading controls and antibodies were used as in B and C. 37 kDa is a molecular weight. E. Multiple conditions for processing were used to rule [file pone.0082021.s004.tif]

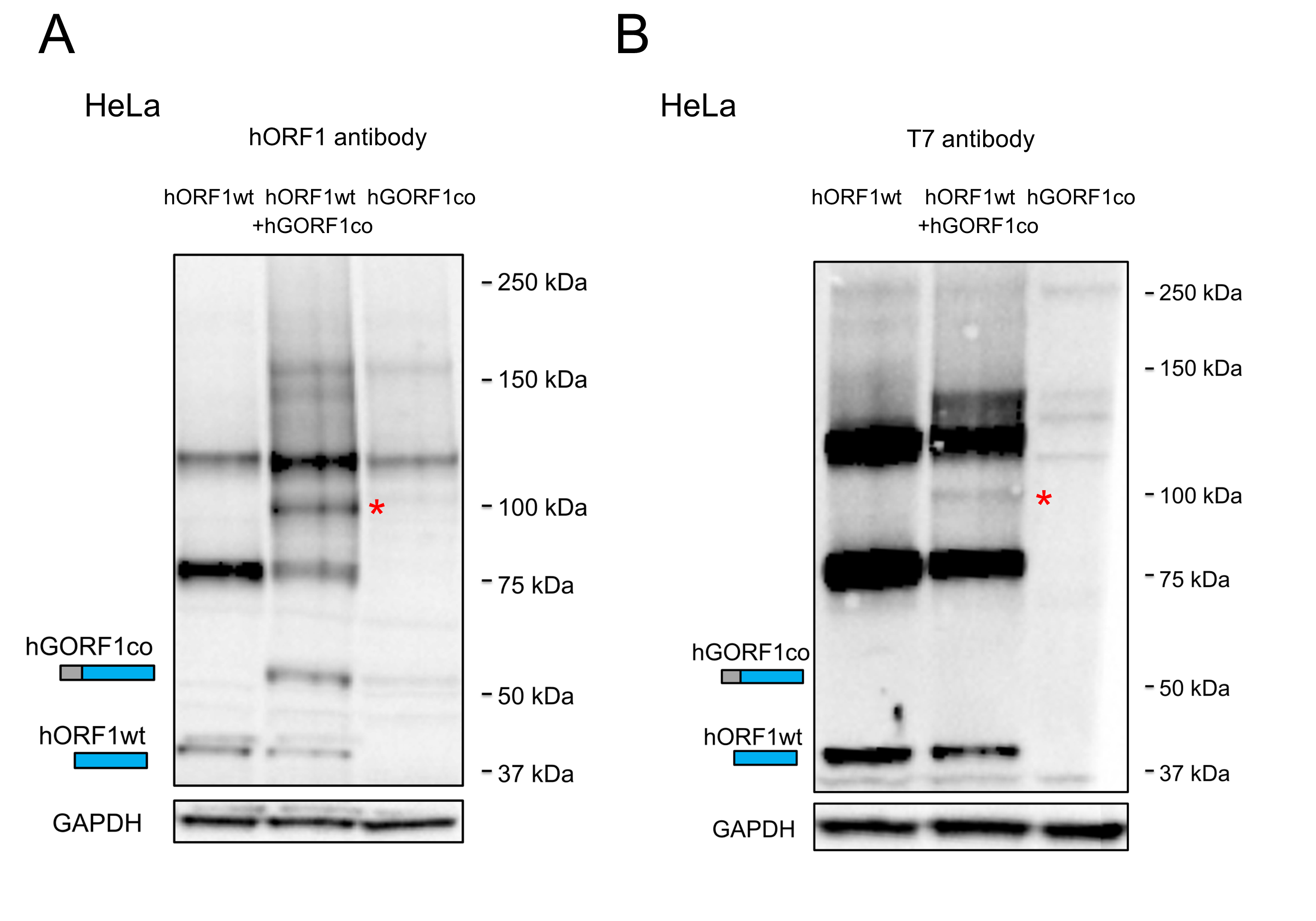

Supplement: Figure S5 — ORF1p heterodimer formation analysis A. Western blot analysis of ORF1p expressed by hORF1wt (blue rectangle labeled hORF1wt) and hGORF1co (grey/blue rectangle labeled hGORF1co) vectors transiently transfected alone or cotransfected in HeLa cells. Red asterisk denotes an ORF1p heterodimer. Proteins are detected with human specific polyclonal antibodies. GAPDH was used as loading control. Molecular weights are indicated on the right in kilodaltons (kDa). B. The same experiment as in A. T7 tag-specific antibodies were used for protein detection. Red asterisk denotes an ORF1p heterodimer. GAPDH was used as loading control. Molecular weights are indicated on the right in kilodaltons (kDa). (TIF) [file pone.0082021.s005.tif]

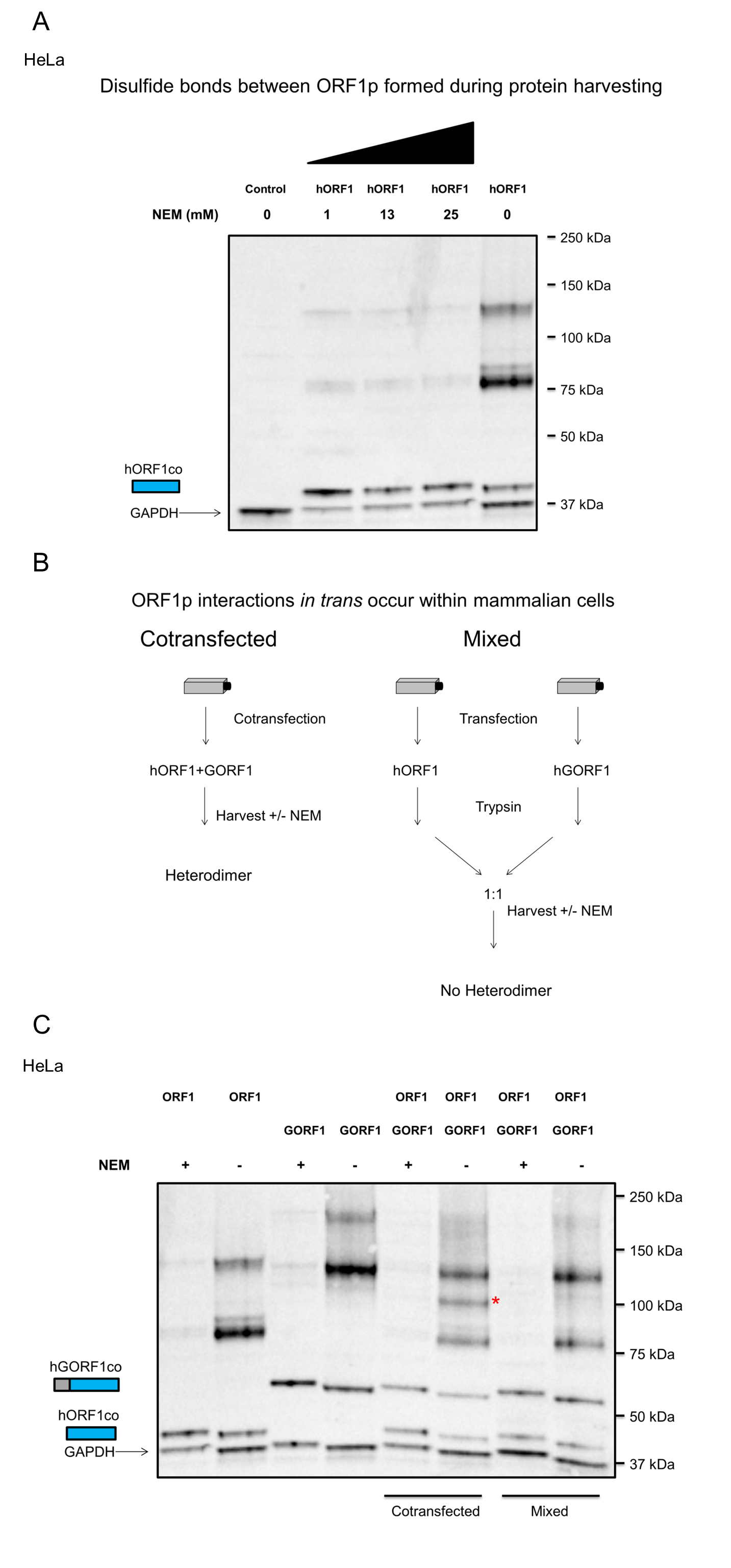

Supplement: Figure S6 — Disulfide bond formation and ORF1p self-interaction. A. Western blot analysis of hORF1co expressed in HeLa cells transiently transfected with hORF1co expression plasmid. Total protein was harvested in the presence of increasing amounts of N-ethylmaleimide (NEM) and analyzed on a non-reducing SDS-PAGE gel. Molecular weights in kilodaltons (kDa) are indicated on the right. B. Schematic of the experimental approach. Total protein was collected with or without NEM (25mM) from HeLa cells cotransfected with hORF1co and hGORF1co expression plasmids (see left, labeled cotransfected) or transfected individually (see right, labeled mixed). The control is designed to determine whether hORF1p and hGORF1p can interact during sample processing. Briefly, cells transfected with either hORF1 or hGORF1 expression plasmids were trypsinized and mixed in equal amounts. Total protein was extracted from the mixture with or without the addition of NEM. No interactions in trans were detected under these experimental conditions. C. Western blot of ORF1p (blue rectangle labeled hORF1co is unfused, grey/blue rectangle labeled hGORF1co is a fusion ORF1 protein) interactions in trans occurring within mammalian cells. Proteins are detected with human-specific ORF1p polyclonal antibodies. GAPDH used as loading control. Molecular weights in kilodaltons (kDa) are shown on the right. Red asterisk is an ORF1p heterodimer. (TIF) [file pone.0082021.s006.tif]

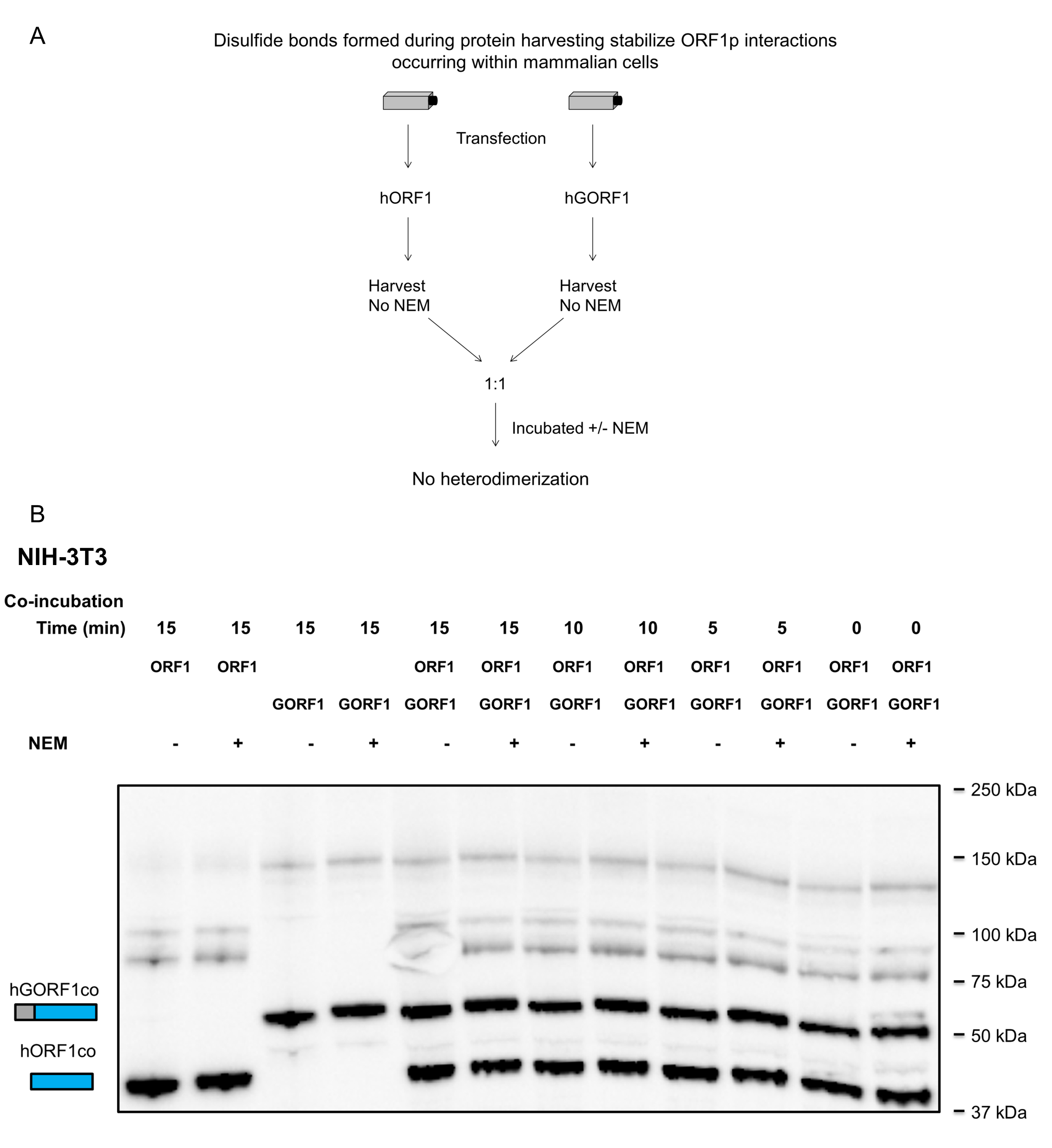

Supplement: Figure S7 — Analysis of ORF1p species as a function of incubation time. A. Schematic of experiment. Cells are transfected individually with either hORF1co or hGORF1co expression plasmids and processed separately. Samples are combined prior to loading on western blot for indicated co-incubation time (see B), with or without NEM (25mM). B. Western blot analysis of ORF1p species formed during protein harvesting detects monomer, dimers, and trimers. No heterodimer formation was observed under these experimental conditions. Proteins are detected with human-specific ORF1p polyclonal antibodies. GAPDH used as loading control. Molecular weights in kilodaltons (kDa) are displayed on the right. (TIF) [file pone.0082021.s007.tif]

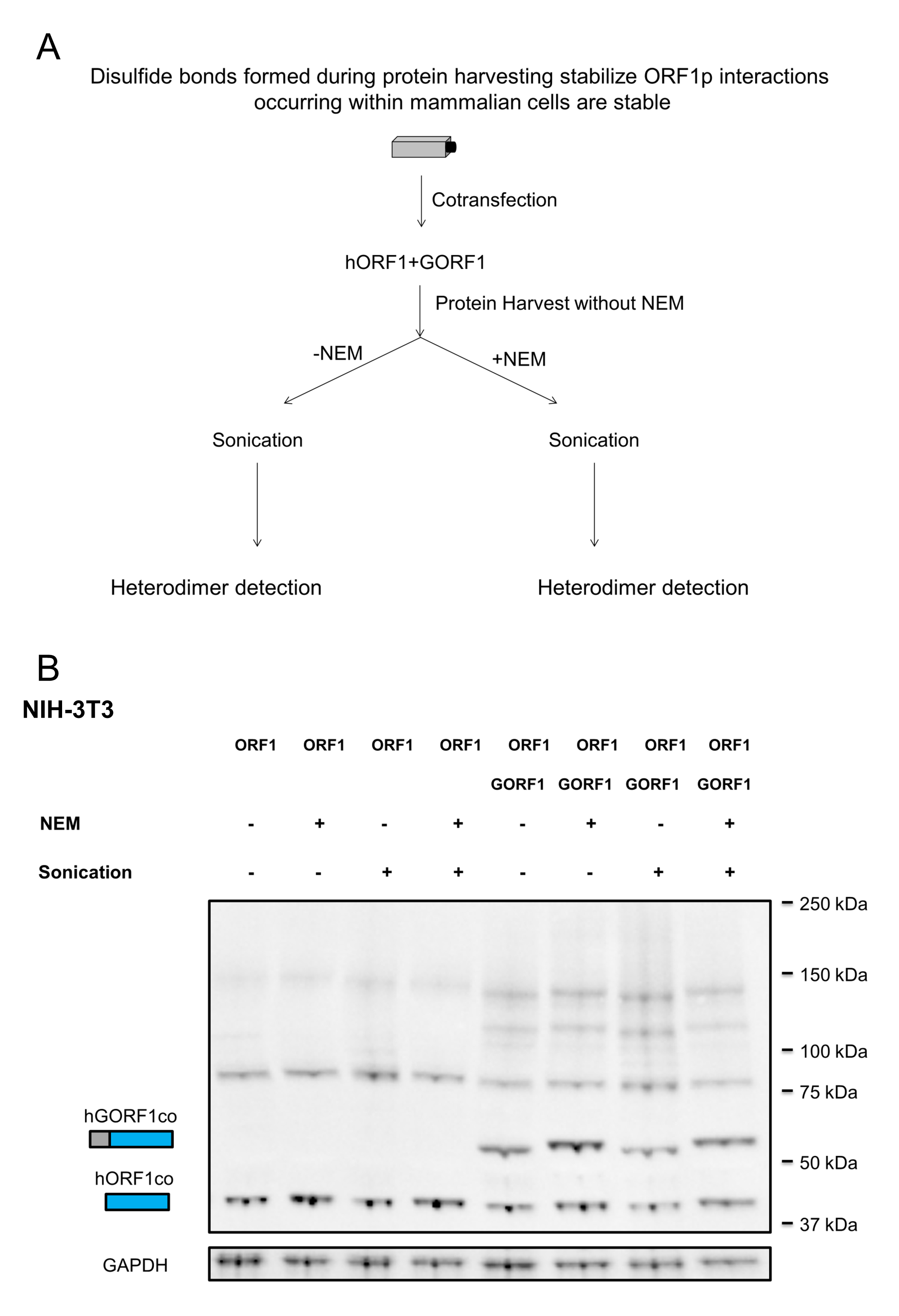

Supplement: Figure S8 — Sonication following protein harvesting does not effect observed ORF1p species. A. Schematic of experiment. Cells are transfected individually or cotransfected with hORF1co and/or hGORF1co, and lysates are harvested without NEM. After processing, samples are split into two tubes, with one tube having 25 mM NEM added and the other with an equivalent amount of ethanol. Samples were then sonicated and fractionated on an SDS-PAGE gel under non-reducing conditions (see materials and methods). B. Western blot analysis of ORF1p species formed during protein harvesting detects heterodimers which are stable regardless of sonication or the presence/absence of NEM. Proteins are detected with human-specific ORF1p polyclonal antibodies. GAPDH used as loading control. Molecular weights in kilodaltons (kDa) are displayed on the right. (TIF) [file pone.0082021.s008.tif]

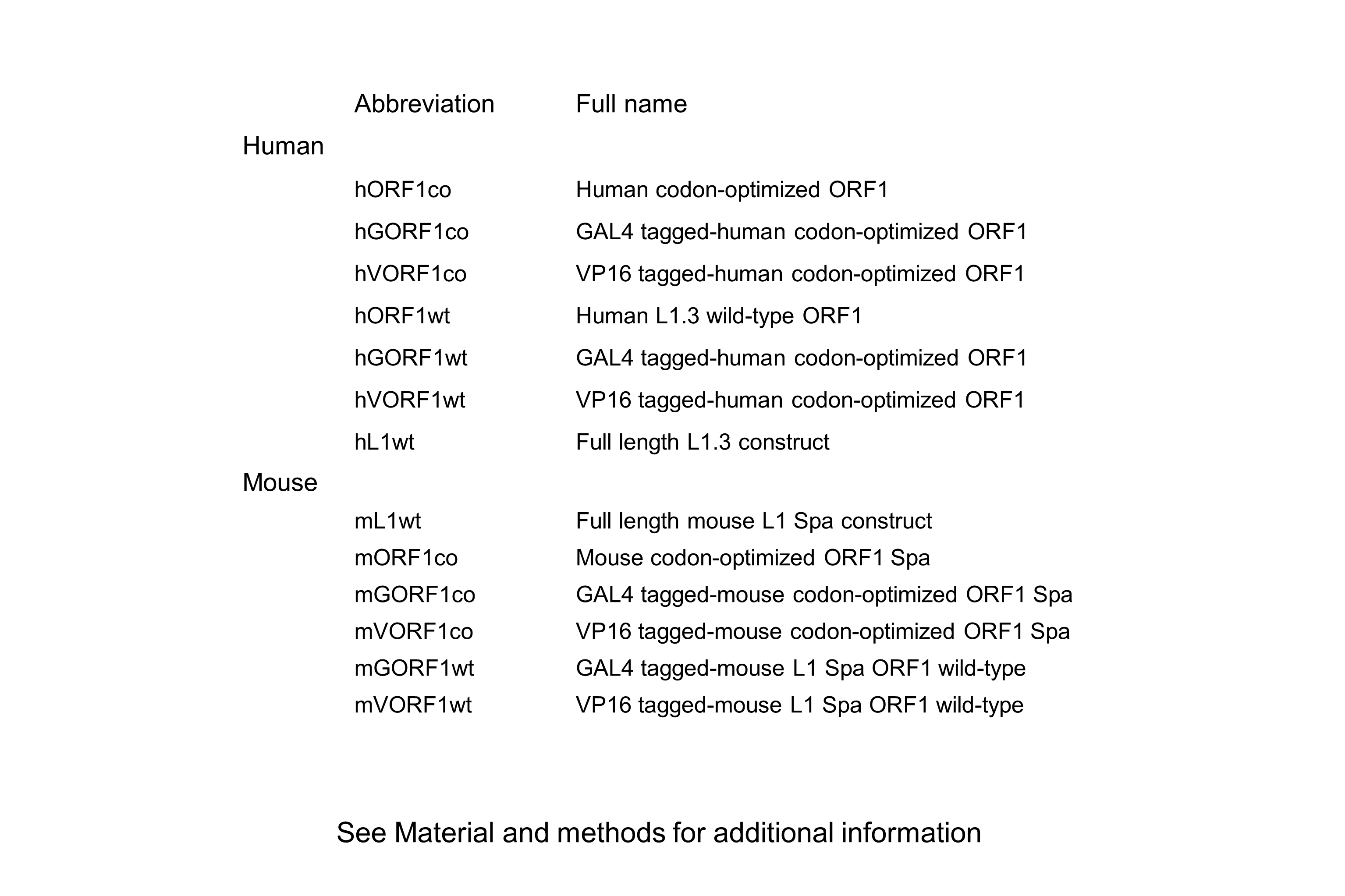

Supplement: Table S1 — Constructs utilized in the study of ORF1p self-interaction. Constructs expressing ORF1p of human and mouse origin are shown. Abbreviations are shown on the left with the parent construct given on the right. (TIF) [file pone.0082021.s009.tif]
